# Taurine Protects C2C12 Myoblasts From Impaired Cell Proliferation and Myotube Differentiation Under Cisplatin-Induced ROS Exposure

**DOI:** 10.3389/fmolb.2021.685362

**Published:** 2021-05-26

**Authors:** Lin Zhou, Ruohan Lu, Caihua Huang, Donghai Lin

**Affiliations:** ^1^ Key Laboratory for Chemical Biology of Fujian Province, MOE Key Laboratory of Spectrochemical Analysis and Instrumentation, College of Chemistry and Chemical Engineering, Xiamen University, Xiamen, China; ^2^ Research and Communication Center of Exercise and Health, Xiamen University of Technology, Xiamen, China

**Keywords:** taurine, C2C12 myoblasts, myotube differentiation, NMR-based metabolomics, ROS, cisplatin

## Abstract

In cancer patients, chemotherapeutic medication induces aberrant ROS (reactive oxygen species) accumulation in skeletal muscles, resulting in myofiber degradation, muscle weakness, and even cachexia, which further leads to poor therapeutic outcomes. Acting as an antioxidant, taurine is extensively used to accelerate postexercise muscle recovery in athletes. The antioxidant effects of taurine have been shown in mature myotubes and myofibers but not yet in myoblasts, the myotube precursor. The proliferation and differentiation ability of myoblasts play a very important role in myofiber repair and regeneration, which is usually impaired during chemotherapeutics in cancer patients as well. Here, we explored the effects of taurine supplementation on C2C12 myoblasts exposed to cisplatin-induced ROS. We found that cisplatin treatment led to dramatically decreased cell viability; accumulated ROS level; down-regulated expressions of MyoD1 (myoblast determination protein 1), myogenin, and MHC (myosin heavy chain); and impaired myotube differentiation in myoblasts. Significantly, taurine supplementation protected myoblasts against cisplatin-induced cell viability decrease, promoted cellular ROS clearance, and, most importantly, preserved the expressions of MyoD1, myogenin, and MHC as well as myotube differentiation ability. We further conducted NMR-based metabolomic analysis to clarify the underlying molecular mechanisms. We identified 14 characteristic metabolites primarily responsible for the discrimination of metabolic profiles between cisplatin-treated cells and normal counterparts, including increased levels of BCAAs (branched-chain amino acids: leucine and isoleucine), alanine, glycine, threonine, glucose, ADP (adenosine diphosphate), phenylalanine, and PC (O-phosphocholine), and decreased levels of lysine, β-alanine, choline, GPC (sn-glycero-3-phosphocholine), and myo-inositol. Evidently, taurine supplementation partially reversed the changing trends of several metabolites (isoleucine, threonine, glycine, PC, β-alanine, lysine, and myo-inositol). Furthermore, taurine supplementation promoted the proliferation and myotube differentiation of myoblasts by alleviating cellular catabolism, facilitating GSH (reduced glutathione) biosynthesis, improving glucose utilization and TCA (tricarboxylic acid) cycle anaplerosis, and stabilizing cellular membranes. Our results demonstrated the protective effects of taurine on cisplatin-impaired myoblasts and elucidated the mechanistic rationale for the use of taurine to ameliorate muscle toxicity in clinical chemotherapy cancer patients.

## Introduction

Skeletal muscle is physiologically essential for human somatic functions such as movement, posture maintenance, heat generation, and breathing (Frontera and Ochala, 2015), which are all energy consuming. As well known, mitochondria possess a central role in energy production through oxidative phosphorylation. Moreover, mitochondria are also the main reactive oxygen species (ROS) generators that produce immensely reactive species by complexes I and III of the electron transport chain (ETC) when there is a leak of electrons (Zorov et al., 2014; Oyewole and Birch-Machin, 2015; Lejri et al., 2019). The inherent motion attribute of skeletal muscles is usually accompanied by the production of oxidative stress, which potentially results in myofiber damage upon untimely clearance (Steinbacher and Eckl, 2015; Magherini et al., 2019; Cheng et al., 2020). Typically in cancer patients, some antineoplastic drugs exhibit high tendencies of muscle toxicity by inducing ROS production (Kodera, 2015; Chen et al., 2016; Kakinuma et al., 2018; Matsuura et al., 2020). For example, cisplatin is a widely used chemotherapeutic drug with side effects including muscle weakness, muscle atrophy, or even cachexia. Long-term cisplatin medication can induce aberrant ROS accumulation in skeletal muscle cells and lead to systemic muscle impairments including promoted muscle catabolism, apoptosis, and autophagy, and cumulated poor therapeutic response and outcome in cancer patients (Sakai et al., 2014; Conte et al., 2020). However, few efficient approaches have been developed for the treatment of cancer patients through directly ameliorating muscle toxicities during chemotherapy.

As a natural antioxidant, taurine is a sulfur-containing β-amino acid that freely exists in most tissues and organs of humans and animals (Froger et al., 2014). In mammalian cells, taurine is primarily taken from the extracellular space by TauT (taurine transporter) (Han and Chesney, 2006; Han et al., 2006) to perform a variety of biological functions, such as detoxification, membrane stabilization, antioxidation, osmoregulation, neuromodulation, and brain and retina development (Militante and Lombardini, 2002; Leon et al., 2009; Schaffer et al., 2009; Schaffer et al., 2010; Froger et al., 2014; Moon et al., 2015; Seidel et al., 2019; Ma et al., 2021). Furthermore, taurine is the most abundant free amino acid in skeletal muscle cells, which serve as the most important pool of taurine in the adult body (Spriet and Whitfield, 2015). Growing evidence has demonstrated that a normal level of taurine is essential for the maintenance of normal muscle function (Matsuzaki et al., 2002; Leon et al., 2009; Schaffer et al., 2010; Spriet and Whitfield, 2015; Thirupathi et al., 2018; Seidel et al., 2019; Thirupathi et al., 2020). Taurine biosynthesis starts with the oxidation of the thiol group of the cysteine, followed by a decarboxylation reaction and spontaneous reaction to form taurine (Huxtable and Lippincott, 1982; Franconi et al., 2006). Unfortunately, the majority of mammals have mostly lost the ability to synthesize taurine and merely rely on nutritional sources (Froger et al., 2014). Therefore, dietary taurine supplementation is indispensable to meet organismal demands. For instance, taurine-containing sports beverages are widely used to speed up muscle recovery in athletes after vigorous exercise (Alford et al., 2001; van den Eynde et al., 2008).

The antioxidant effects of taurine have been shown in mature myotubes and myofibers (Stacchiotti et al., 2014; Thirupathi et al., 2018; Seidel et al., 2019; Thirupathi et al., 2020) but not yet in myoblasts which are capable of proliferating, differentiating, and fusing to form myofibers upon muscle regeneration stimulus. Significantly, myoblasts can also be impaired in cancer patients after chemotherapeutic medication, resulting in the dysfunction of muscle regeneration. Given that myoblasts play a critical role in the post-damage regeneration of myofibers, we investigated in this study the effects of taurine supplementation on murine C2C12 myoblasts impaired by cisplatin treatment. We found that taurine supplementation could protect myoblasts against cisplatin-induced cell viability decrease, promote cellular ROS clearance, and preserve myotube differentiation ability. Moreover, we found that taurine supplementation could partially reverse the changing trends of several metabolites in cisplatin-treated myoblasts. We further performed NMR-based metabolomic analysis to address the underlying molecular mechanisms. Our results indicate that taurine supplementation may be a promising strategy for ameliorating muscle toxicity induced by chemotherapeutics.

## Materials and Methods

### Reagents

Taurine (#T0625), cisplatin (#P4394), and H_2_DCFDA (#287810) were purchased from Sigma-Aldrich. The MDA assay kit (#S0131S) was purchased from Beyotime Biotechnology (China). Two primary antibodies were used: MyoD1 (ab64159, Abcam), Myogenin (67082-1-lg, Proteintech), MHC (3405S, Cell Signaling Technology), AKT (10176-2-AP, Proteintech), p-AKT (AP3434A, ABGENT), caspase 3 (19677-1-AP, Proteintech), GAPDH (10494-1-AP, Proteintech).

### Cell Culture and Experimental Design

Murine C2C12 myoblasts were provided by Stem Cell Bank, Chinese Academy of Sciences, China (Shanghai, CHN). C2C12 myoblasts were grown in DMEM culture medium supplemented with 100 units/mL penicillin, 100 μg/mL streptomycin, and 10% fetal bovine serum (FBS, HyClone). The differentiation medium (DM) was prepared from DMEM containing 2% heat-inactivated horse serum. Cells were cultured in a humidified atmosphere of 5% CO_2_ at 37°C. A brief illustration of the experimental design is shown in Supplementary Figure S1. Cisplatin was dissolved in DMSO to obtain a 5-mM stock solution and administered to the cells at a final concentration of 10 μM for 36 h in the cisplatin-treated (CIS) group of C2C12 myoblasts (Supplementary Figure S1A). Taurine (5 mM) was added in DMEM for 12 h before cisplatin treatment in the taurine-supplemented (TAU) group of C2C12 myoblasts (Supplementary Figure S1A). Control experiments were conducted with the equivalent amount of DMSO in the normal control (NOR) group of the cells. For in vitro myotube differentiation experiments (Supplementary Figure S1B), C2C12 myoblasts were used within 10 generations of culture. When the cells reached 85%–90% confluence after 24 h culture, myotube differentiation was induced by incubation in the DM for 96 h. Similarly, the same treatment procedures of cisplatin and taurine were carried out in the corresponding groups, and the DM was changed every 24 h.

### ROS Measurements With the H_2_DCFDA Probe

Intracellular ROS levels were measured with the fluorescent cell-permeable probe H_2_DCFDA (Lyublinskaya et al., 2017). Briefly, C2C12 myoblasts (2 × 10^5^ cells/well) were cultured in DMEM as described above, then washed three times with PBS, followed by incubation with H_2_DCFDA (5 μM) for 30 min at 37°C. Subsequently, the cells were washed again with PBS three times. Fluorescence images were acquired using a confocal microscope (Leica DM 4000B, Germany) with a 20× objective lens (λ_*ex*_ = 508 nm, λ_*em*_ = 535 nm). Intracellular ROS levels were determined by the fluorescence intensity of H_2_DCFDA detected by flow cytometry.

### Lipid Peroxidation Assessed by the MDA Assay

Cell samples were subjected to the MDA assay as described in the Lipid Peroxidation MDA Assay Kit (Beyotime, Nantong, China). MDA levels were measured at a wavelength of 530 nm by multimode microplate readers (POLARstar Omega, BMG LABTECH GmBH, Germany), which reflected the lipid peroxidation induced by ROS.

### Cell Viability Measurements Using the MTS Assay

Cell viability measurements were performed using a CellTiter 96 AQueous One Solution Cell Proliferation Assay Kit (Promega, Madison, United States) according to the recommendations of the manufacturer. Cells were seeded at a density of 5 × 10^3^ cells per well in 96-well plates and cultured as described above. Equivalent volumes of vehicle culture media were treated as controls. Then, 20 μL of MTS was added to each well containing 100 μL of the culture medium, and the cells were incubated in the dark at 37°C for 3 h before measuring the absorbance of formazan at a wavelength of 490 nm on a multimode microplate reader (POLARstar Omega, BMG LABTECH GmBH, Germany). Morphologies of the C2C12 myoblasts were examined using an inverted microscope. Pictures were taken at randomly selected visual points.

### Protein Expression Measurements

Intracellular proteins of C2C12 myoblasts were isolated using RIPA lysis buffer with protease inhibitor and phosphorylation protease inhibitor cocktails (Thermo Fisher). The homogenates were then sonicated for 35 s and centrifuged (11,000 *g* for 10 min at 4°C) to remove the debris. The supernatants were collected, and protein concentrations were determined using a BCA Protein Assay Kit (Beyotime Biotechnology). Then, the denatured samples were subjected to SDS-PAGE and transferred to PVDF membranes (GE Healthcare) for immunoblotting analysis. After blocking with 5% nonfat milk in Tris-buffered saline containing 0.1% Tween 20 for 1 h, the membranes were probed with corresponding antibodies (MyoD1, Abcam, ab64159; GAPDH, ProteinTech, 10494-1-AP). Proteins were visualized by enhanced chemiluminescence using horseradish peroxidase–conjugated antibodies, and band densities were quantified by the ImageJ software.

### Myotube Morphological Assessment

C2C12 myotubes were fixed with pre-cold methanol for 30 s, stained with 0.1% crystal violet solution for 10 min, and rinsed with distilled water before taking microscopic photographs. Myotube differentiation was assessed on representative microscopic images at 20× magnification for morphological analysis (Motic, AE31E, Xiamen, CHN). Images were subsequently used for quantitative measurement of myotube number and area by ImageJ (Java software, National Institutes of Health, United States). Myotube area was determined by carefully tracing around myotube structures on each image produced by the microscope. The total area of myotubes was normalized by nuclear numbers.

### Cellular Metabolite Extraction

Aqueous metabolites were extracted from C2C12 myoblasts for NMR-based metabolomic analysis according to the protocol described previously (Beckonert et al., 2007; Cui et al., 2019a; Cui et al., 2019b). For preparation of intracellular metabolite samples, about 5 × 10^6^ cells in each culture dish were harvested. A mixture of methanol, chloroform, and water at a volume ratio of 4:4:2.85 was applied in dual-phase extraction for extracting intracellular metabolites. Afterward, the upper polar cell extracts were lyophilized and suspended in 550 μL of NMR buffer, in which a final concentration of 0.1 mM sodium 3-(trimethylsilyl) propionate-2,2,3,3-d4 (TSP) was added for providing the NMR chemical shift reference (δ 0.00). All samples were centrifuged and transferred into 5-mm NMR tubes for the following NMR experiments.

### NMR Measurements

All NMR experiments were performed at 25°C on a Bruker Avance III 850 MHz spectrometer (Bruker BioSpin, Germany) equipped with a TCI cryoprobe. One-dimensional (1D) ^1^H spectra were recorded on aqueous cell extracts using the pulse sequence NOESYGPPR1D [RD-G1-90°-t1-90°-τ_m_-G2-90°-ACQ] with water suppression during the relaxation delay and mixing time. t1 was a short delay (10 μs), and τ_m_ was the mixing time (10 m). Pulsed gradients G1 and G2 were used to improve water suppression quality. A total of 32 transients were collected into 64 K data points using a spectral width of 20 ppm with an acquisition time (ACQ) of 2.66 s, using an additional relaxation delay (4s). Based on the 1D ^1^H spectra, metabolites were identified using a combination of Chenomx NMR Suite software (version 8.3, Chenomx Inc, Canada), the Human Metabolome Data Base (HMDB, http://www.hmdb.ca/), and relevant published references (Xu et al., 2017; Liu et al., 2018). To confirm the resonance assignments of the metabolites, two-dimensional (2D) ^1^H-^13^C heteronuclear single quantum coherence (HSQC) spectra and ^1^H–^1^H total correlation spectroscopy (TOCSY) spectra were recorded on selected NMR samples.

### NMR Data Processing

Free induction delay (FID) signals were processed by applying an exponential function with a line-broadening factor of 0.3 Hz before Fourier transformation, followed by manual phasing and baseline correction using MestReNova software (version 9.0, Mestrelab Research S.L., Spain). Chemical shift correction was referenced to the methyl group of TSP at 0 ppm. Spectral regions of δ 5.2–4.65 (water resonance) were removed from the spectra, then the regions of δ 9.5–0.8 were binned by 0.001 ppm for statistical analysis. Peak integrals for each NMR spectrum were normalized by both the TSP peak integral and the weight of the tissue slice. The relative intensity of each metabolite was calculated based on both the relative integral of singlet or nonoverlapped peaks in each NMR spectrum and the proton number contained in the metabolite. The relative intensity of the metabolite was used to present its relative level and was represented as mean ± standard deviation (SD).

### Multivariate Analysis and Univariate Analysis

Multi variate statistical analysis was conducted using SIMCA-P^+^ 14.1 software (MKS Umetrics AB, Sweden). Pareto scaling was applied to increase the magnitudes of low-abundance metabolites without significant amplification of noise. To reveal metabolic separation and show clusters among samples, unsupervised principal component analysis (PCA) was performed. Furthermore, supervised partial least-squares discriminant analysis (PLS-DA) was subsequently performed to obtain a better separation between metabolic profiles. The PLS-DA model was cross-validated to evaluate its robustness by a response permutation test (RPT) with 200 cycles. Then, significant metabolites were identified with the criterion of variable importance in projection (VIP) > 1 calculated from the PLS-DA model. In addition, hierarchical cluster analysis (HCA) was conducted using MetaboAnalyst 4.0 webserver (http://www.metaboanalyst.ca/) with Spearman distance measure and Ward clustering algorithm to further confirm the metabolic clusters.

One-way analysis of variance (ANOVA) followed by Tukey’s multiple comparison test was performed to quantitatively compare metabolite levels among the three groups of intracellular extracts with SPSS 19 software. Differential metabolites were identified with the criterion of statistical significance (*p*) <0.05, which were combined with significant metabolites identified from the PLS-DA to give characteristic metabolites.

### Metabolic Pathway Analysis

Significantly altered metabolic pathways were identified based on the relative levels of the assigned metabolites with a combination of metabolite set enrichment analysis (*p* < 0.05) and pathway topological analysis (pathway impact value >0.2). The metabolic pathway analysis was performed on MetaboAnalyst 4.0 webserver (https://www.metaboanalyst.ca). This webserver was also used to obtain the heat-map plots of relative levels of the metabolites, and Pearson’s correlation coefficients of intracellular ROS/MDA levels and cell viabilities with intracellular levels of the characteristic metabolites.

### General Statistical Analysis

Experimental data were expressed as means ± SD. For quantitative comparison between two groups, data were analyzed by Student’s *t*-test using GraphPad Prism software (version 6.01, La Jolla, USA). For pairwise comparisons among three or more groups, data were analyzed by ANOVA, followed by Tukey’s multiple comparison tests with SPSS 19. Statistical significances were as follows: *p* > 0.05 (NS), *p* < 0.05 (*), *p* < 0.01 (**), *p* < 0.001 (***), and *p* < 0.0001 (****).

## Results

### Taurine-Facilitated ROS Clearance in C2C12 Myoblasts

**FIGURE 1:**
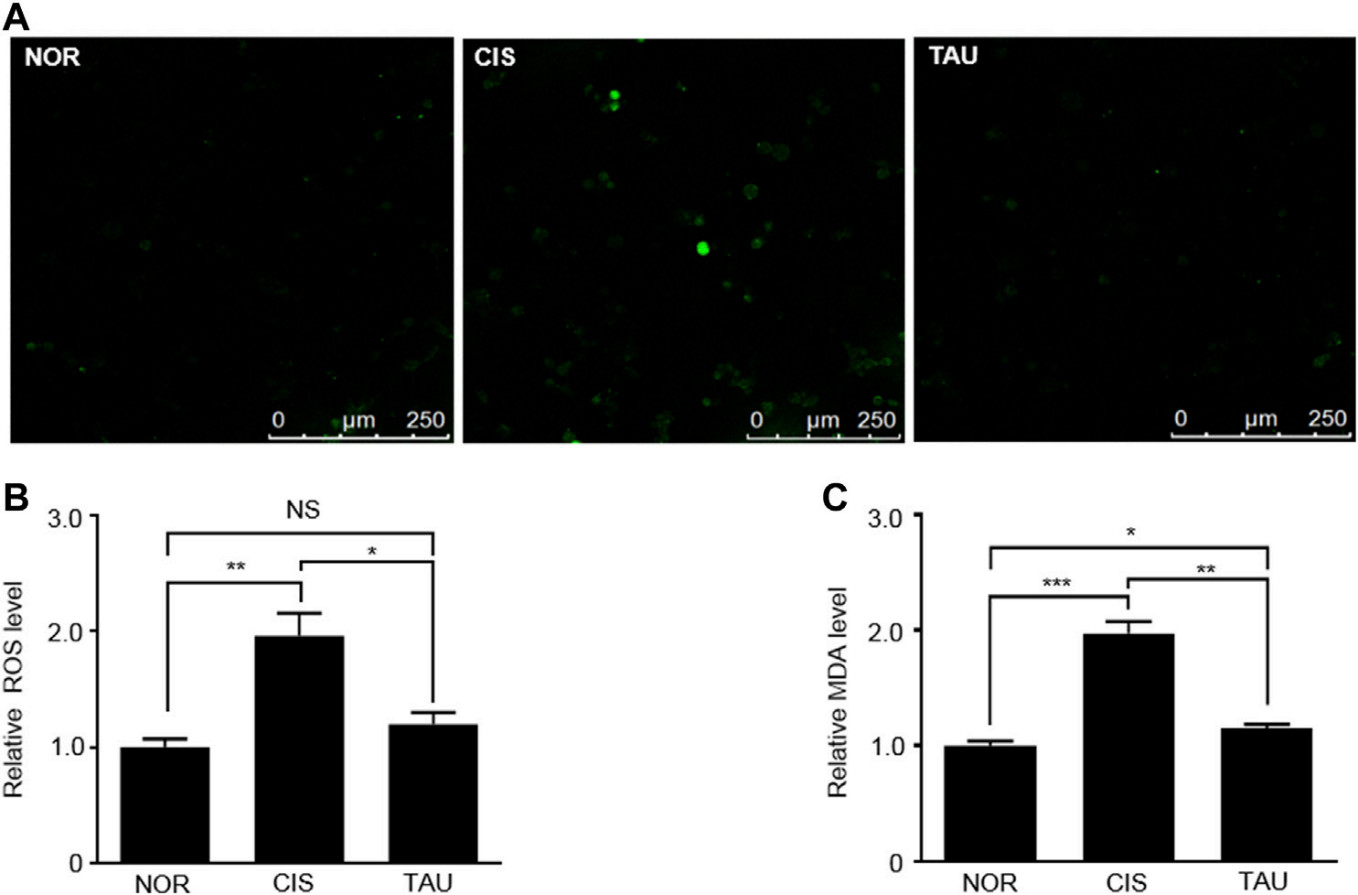
Taurine supplementation promoted the clearance of cisplatin-induced ROS in C2C12 myoblasts. **(A)** Representative confocal microscopy image of H_2_DCFDA-stained normal control (NOR) myoblasts, cisplatin-treated (CIS) myoblasts, and taurine-supplemented (TAU) myoblasts. Scale bar, 50 µm. **(B)** Intracellular ROS levels determined by oxidized H_2_DCFDA fluorescence signals measured by flow cytometry in C2C12 myoblasts (*n* = 6). **(C)** Intracellular MDA levels measured by the MDA assay at 530 nm (*n* = 6). The MDA level was used to reflect the lipid peroxidation induced by ROS.

Cisplatin is a well-known chemotherapeutic drug for solid tumors and leukemia with muscle toxicity, and long-term cisplatin medication can induce aberrant ROS accumulation in muscle cells and result in systemic muscle impairments (Sakai et al., 2014; Conte et al., 2020). We measured intracellular ROS levels in the three groups of C2C12 myoblasts with the H_2_DCFDA (2′,7′-dichlorofluorescin diacetate) probe (Figures 1A,B and Supplementary Figure S7). The CIS myoblasts showed a one-fold increase in the ROS level relative to the NOR myoblasts (1.96 ± 0.37 vs. 1.0 ± 0.13), indicating that cisplatin treatment induced aberrant ROS accumulation (Figure 1B). Significantly, taurine supplementation eliminated the accumulated intracellular ROS, as indicated by the ROS level of 1.2 ± 0.17 in the TAU myoblasts relative to the CIS myoblasts (Figure 1B).

Furthermore, we measured the MDA (malondialdehyde) levels using the MDA assay, which reflected the ROS-induced lipid peroxidation. Similarly, the CIS myoblasts displayed a one-fold increase in the MDA level relative to the NOR myoblasts (1.97 ± 0.17 vs. 1.0 ± 0.07), and taurine supplementation evidently inhibited MDA generation, as indicated by the MDA level of 1.15 ± 0.06 in the TAU myoblasts relative to the CIS myoblasts (Figure 1C). These results exhibited that taurine supplementation greatly neutralized the cisplatin-induced ROS and facilitated ROS clearance in cisplatin-impaired C2C12 myoblasts.

### Taurine-Protected C2C12 Myoblasts Against Cisplatin-Induced Cell Viability Decrease

**FIGURE 2:**
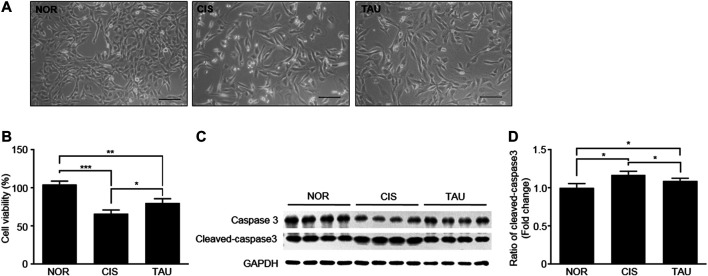
Taurine supplementation protected C2C12 myoblasts against cisplatin-induced cell viability decrease. **(A)** Representative morphological images of the three groups of C2C12 myoblasts (NOR, CIS, and TAU). Scale bar, 100 μm. **(B)** Percentage of cell viability measured by the MTS assay (*n* = 6). **(C, D)** Expression of caspase-3 and cleaved-caspase-3 in the three groups of C2C12 myoblasts (*n* = 8).

We measured cell viabilities of the three groups of C2C12 myoblasts using the MTS (3-(4,5-dimethylthiazol-2-yl)-5-(3-carboxymethoxyphenyl)-2-(4-sulfophenyl)-2H-tetrazolium, inner salt) assay (Figure 2A and Supplementary Figure S8). The CIS myoblasts showed a significant decrease in cell viability compared with the NOR myoblasts (0.66 ± 0.05 vs. 1.04 ± 0.04), which was partially restored in the TAU myoblasts as indicated by the cell viability of 0.8 ± 0.03 relative to the CIS myoblasts (Figure 2B). Previous studies have reported that cisplatin treatment can induce apoptosis in various kinds of cell lines (Q. Wang et al., 2019; Z. Wang et al., 2019; Chen et al., 2019; Liu et al., 2019). Consistently, we detected the upregulated expression of an apoptotic marker cleaved caspase-3 in CIS myoblasts, which indicated that cisplatin treatment induced apoptosis in C2C12 myoblasts (Figures 2C,D). Thus, cisplatin treatment not only inhibited cellular proliferation but also induced apoptosis, as reflected by the decreased viable cell population in CIS myoblasts. Significantly, taurine supplementation inhibited cisplatin-induced apoptosis, as indicated by the decreased expression of cleaved caspase-3 (Figures 2C,D). These results showed that taurine supplementation evidently protected C2C12 myoblasts against cisplatin-induced cell viability decrease and significantly promoted cisplatin-impaired cellular proliferation and eliminated cisplatin-induced cellular apoptosis.

### Taurine-Enhanced Cisplatin-Impaired Myotube Differentiation Ability of C2C12 Myoblasts

**FIGURE 3:**
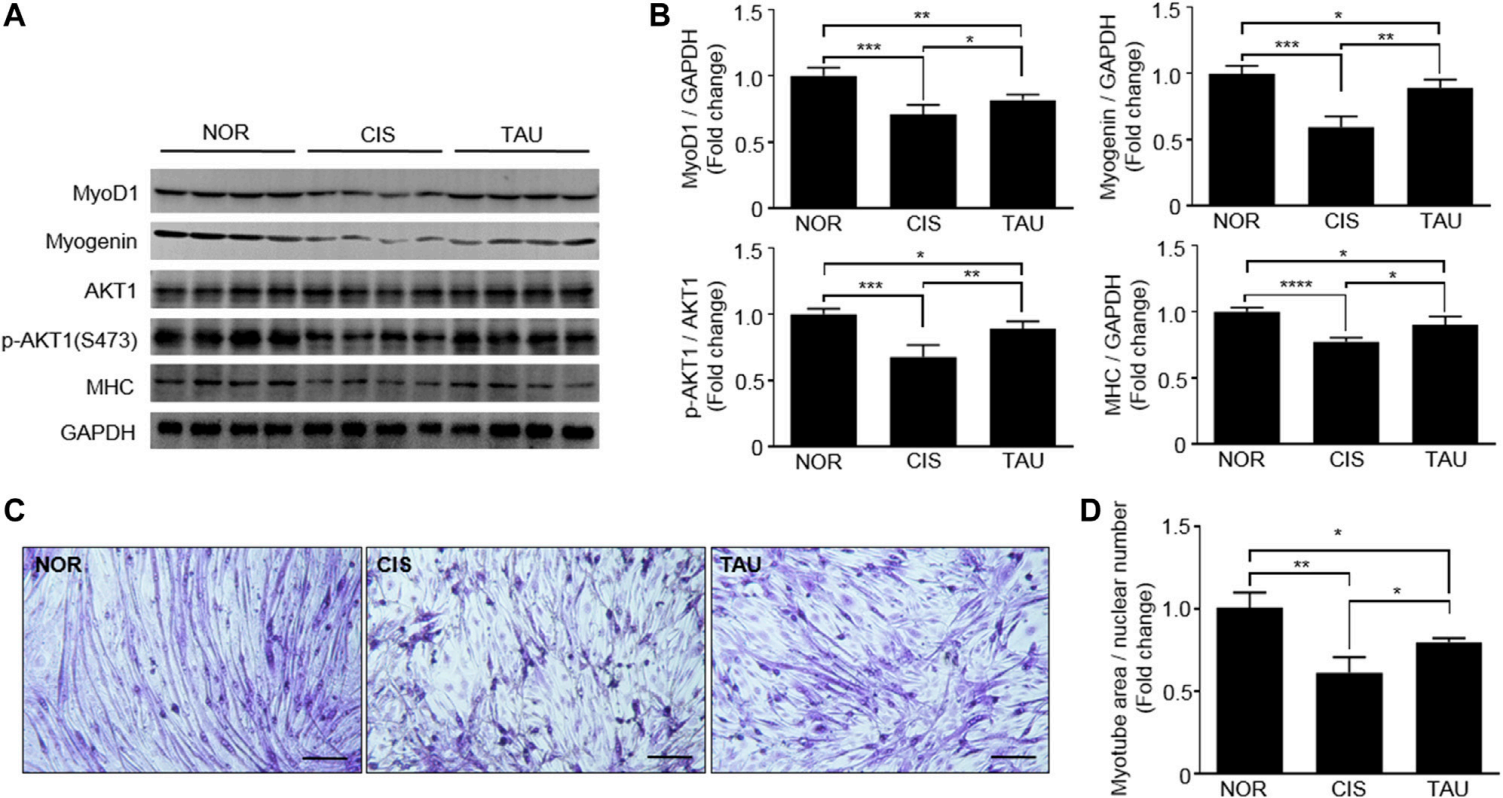
Taurine supplementation preserved the myotube differentiation ability of C2C12 myoblasts. **(A, B)** Expression of MyoD1, myogenin, AKT1, phospho-AKT1 (ser473), and MHC in the three groups of C2C12 myoblasts (*n* = 8). **(C)** Representative morphological images of normal control (NOR) myotubes, cisplatin-treated (CIS) myotubes, and taurine-supplemented (TAU) myotubes (20×). Scale bar, 100 µm. **(D)** Relative myotube area. All experiments were conducted on three separate occasions (*n* = 3); 4–5 morphological images per condition in duplicate were analyzed for each occasion. The total area of myotubes was normalized to nuclear numbers.

Regarding muscle impairments, the differentiation ability of myoblasts is highly critical for muscle repair and regeneration (Wernig, 2003; Chargé and Rudnicki, 2004; Dumont et al., 2015; Frontera and Ochala, 2015). Both MyoD and myogenin dominate downstream genes involved in the formation and maintenance of different mature muscle fibers (Asakura et al., 1993; Zammit, 2017; Ganassi et al., 2020). Myotube differentiation process is closely associated with the regulation of muscle-specific gene expression, which is followed by myofiber protein synthesis. Several proteins, including MyoD, myogenin, myosin heavy chain (MHC), and AKT, are involved in the myotube differentiation process (Buckingham and Rigby, 2014). To characterize the effects of both cisplatin treatment and taurine supplementation on the myotube differentiation ability of C2C12 myoblasts, we measured MyoD1, myogenin, MHC, and AKT1 expressions in the three groups of myoblasts. The CIS myoblasts showed significantly decreased expressions of MyoD1, myogenin, and MHC, and a decreased ratio of p-AKT1 (ser473)/AKT1 compared to the NOR myoblasts, implying that cisplatin treatment significantly declined the myotube differentiation ability of the myoblasts (Figures 3A,B). The TAU myoblasts exhibited that taurine supplementation partially restored cisplatin-decreased expressions of MyoD1, myogenin, and MHC, and significantly increased the ratio of p-AKT1 (ser473)/AKT1 in C2C12 myoblasts (Figures 3A,B). Furthermore, we conducted in vitro myotube differentiation experiments to directly observe the effects of both cisplatin treatment and taurine supplementation on myotube formation of C2C12 myoblasts (Supplementary Figure S1B). Cisplatin treatment dramatically decreased the myotube area of CIS myotubes relative to NOR myotubes, while taurine supplementation recovered the myotube area of TAU myotubes decreased by cisplatin treatment (Figures 3C,D). To some extent, taurine supplementation preserved the myotube differentiation ability of C2C12 myoblasts impaired by cisplatin treatment.

### NMR Spectra of Aqueous Extracts of C2C12 Myoblasts

**FIGURE 4:**
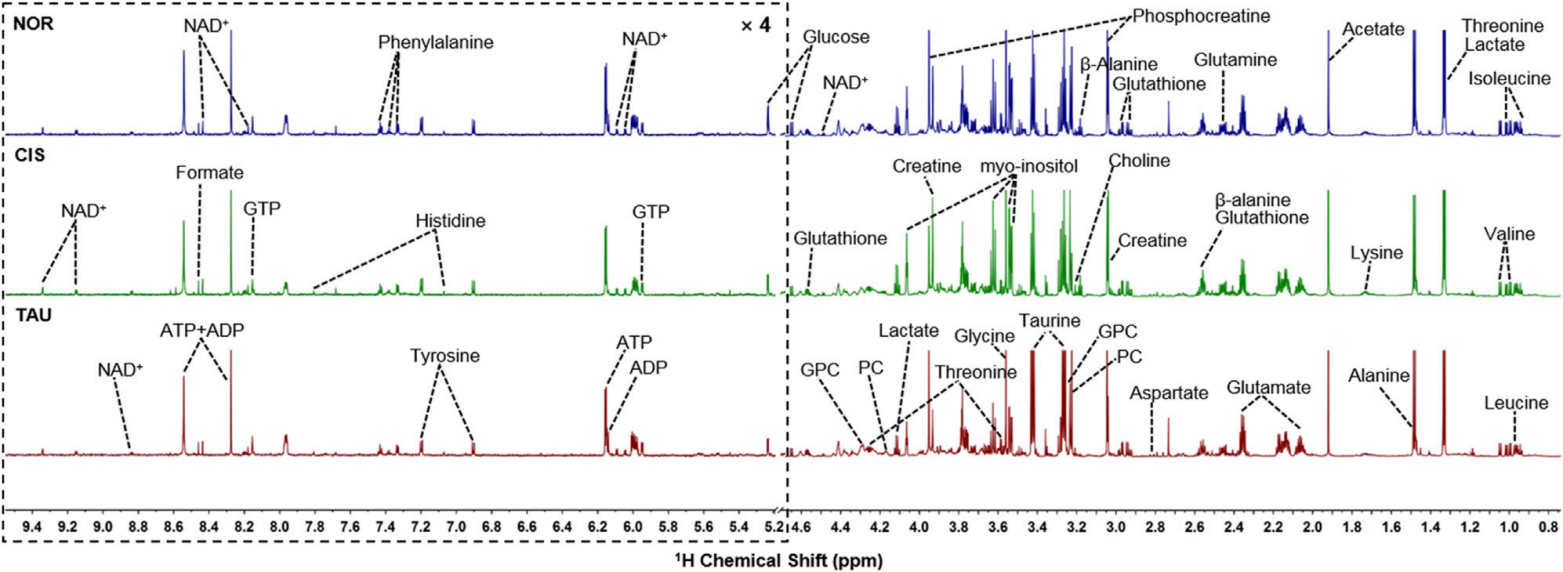
Typical 850 MHz ^1^H NMR spectra recorded on aqueous extracts derived from the three groups of C2C12 myoblasts. Vertical scales are kept constant in all the ^1^H spectra. Spectral regions of 0.8–4.6 ppm and 5.2–9.5 ppm are shown, and the water region of 4.6–5.2 ppm was removed. The region of 5.2–9.5 ppm has been magnified 4 times compared to another region of 0.8–4.6 ppm for the purpose of clarity. Identified metabolites are shown in Table 1. Abbreviations: PC, O-phosphocholine; GPC, sn-glycerol-3-phosphocholine; GTP, guanosine triphosphate; ATP, adenosine triphosphate; ADP, adenosine diphosphate; NAD^+^, nicotinamide adenine dinucleotide.

We conducted NMR-based metabolomic analysis to address the molecular mechanisms underlying the protective effects of taurine supplementation on cisplatin-impaired C2C12 myoblasts. Figure 4 shows the typical 850 MHz ^1^H NMR spectra recorded on aqueous extracts derived from the NOR, CIS, and TAU groups of C2C12 myoblasts. Collectively, 30 metabolites were identified by a combination of Chenomx NMR Suite, the HMDB, and previously published literature (Table 1). As expected, the TAU group exhibited an intracellular level of taurine remarkably higher than the NOR and CIS groups, owing to the supplemented taurine in the culture medium (Supplementary Figure S2). The resonance assignments of the metabolites were confirmed using 2D ^1^H-^13^C HSQC and ^1^H–^1^H TOCSY spectra (Supplementary Figure S3, S4).

**TABLE 1:** Identified metabolites in ^1^H NMR spectra of aqueous extracts derived from C2C12 myoblasts.

NO.	Metabolites	δ ^1^H (ppm) and multiplicity	Moieties
1	Leucine	0.96(d), 0.97(d), 1.69(m), 1.70(m), 1.73(m), 3.73(m)	α-CH_3_, α-CH_3_, γ-CH, β-CH_2_, α-CH
2	Isoleucine	0.94(t), 1.01(d), 1.21(m), 1.42(m), 2.00(m), 3.67(d)	δ-CH_3_, γ-CH_3_, half γ-CH_2_, half γ-CH_2_, β-CH, α-CH
3	Valine	0.99(d), 1.05(d), 2.26(m), 3.60(d)	γ-CH_3_, γ-CH_3_, β-CH, α-CH
4	Alanine	1.47(d), 3.78(q)	β-CH_3_, α-CH
5	Lysine	1.43(m), 1.50(m), 1.73(m), 1.89(m), 1.92(m), 3.02(t), 3.75(t)	γ-CH_2_, half γ-CH_2_, δ-CH_2_, β-CH_2_, ε-CH_2_, α-CH
6	Acetate	1.91(s)	CH_3_
7	Glutamate	2.08(m), 2.12(m), 2.34(m), 2.37(m), 3.75(m)	half β-CH_2_, half β-CH_2_, half γ-CH_2_, half γ-CH_2_, α-CH
8	Glutamine	2.13(m), 2.45(m), 3.77(t)	γ-CH_2_, β-CH_2_, α-CH
9	Glutathione	2.15(m), 2.55(m), 2.96(m), 3.77(m), 4.56(m)	β-CH_2_, γ-CH_2_, CH_2_-SH, α-CH&CH_2_-NH, CH-NH
10	Aspartate	2.68(dd), 2.81(dd), 3.90(dd)	β-CH_2_, α-CH
11	β-Alanine	2.54(t), 3.17(t)	CH_2_, CH_2_
12	Choline	3.20(s), 3.50 (dd), 4.03(t)	N-(CH_3_)_3_, N-CH_2_, CH_2_OH
13	PC	3.22(s), 3.60(t), 4.18(m)	N-(CH_3_)_3_, N-CH_2_, CH_2_OH
14	GPC	3.23(s), 3.60(dd), 3.68(dd), 3.87(m), 3.94(m), 4.33(m)	N-(CH_3_)_3_, half ^1^CH_2_, ^2^CH_2_, half ^1^CH_2_, half ^3^CH_2_, half ^3^CH_2_, ^1^CH_2_
15	Glycine	3.57(s)	α-CH_2_
16	Creatine	3.04(s), 3.93(s)	N-CH_3_, α-CH_2_
17	Phosphocreatine	3.05(s), 4.05(s)	N-CH_3_, CH_2_
18	Myo-inositol	3.28(t), 3.53 (dd), 3.63(t), 4.07(t)	^2^CH,^4,6^CH,^1,3^CH, ^5^CH
19	Lactate	1.33(d), 4.11(q)	β-CH_3_, α-CH
20	Taurine	3.24(t), 3.41(t)	^1^CH_2_, ^2^CH_2_
21	Threonine	1.31(d), 3.59(d), 4.25(m)	γ-CH_2_, β-CH
22	Glucose	β(3.24 (dd), 3.48(t), 3.90 (dd)), α(3.54 (dd), 3.71(t), 3.72 (dd), 3.83(m))	β(H_2_, H_3_, H_5_), α(H_2_, H_3_, H_6_)
23	ADP	6.13 (d), 8.27 (s), 8.58 (s)	NH_2_, δ-CH, ^2^CH
24	ATP	6.14 (d), 8.27 (s), 8.58 (s)	NH_2_, δ-CH, ^2^CH
25	Histidine	7.06(s), 7.85(s)	^5^CH, ^2^CH
26	Tyrosine	3.05(dd), 3.19(dd), 6.92(d), 7.19(d)	half β-CH_2_, half β-CH_2_, β-CH, α-CH
27	Phenylalanine	3.12(dd), 3.30(dd), 3.99(dd), 7.33(d), 7.37(t),7.43(t)	α-CH, half β-CH_2_, half β-CH_2_, α-CH, β-CH, γ-CH
28	GTP	5.92 (d), 8.1 (s)	CH, CH
29	Formate	8.46(s)	CH
30	NAD^+^	6.03(d), 6.08(s), 8.16(s), 8.20(m), 8.41(s), 8.82(d), 9.13(d), 9.32(s)	NH_2_, NH_2_(CO), δ-CH, β-CH, ^2^CH, γ-CH, α-CH

Multiplicity: s, singlet; d, double; t, triplet; q, quartet; m, multiple; dd, double of double.

### Multivariate Data Analysis of Metabolic Profiles of C2C12 Myoblasts

**FIGURE 5:**
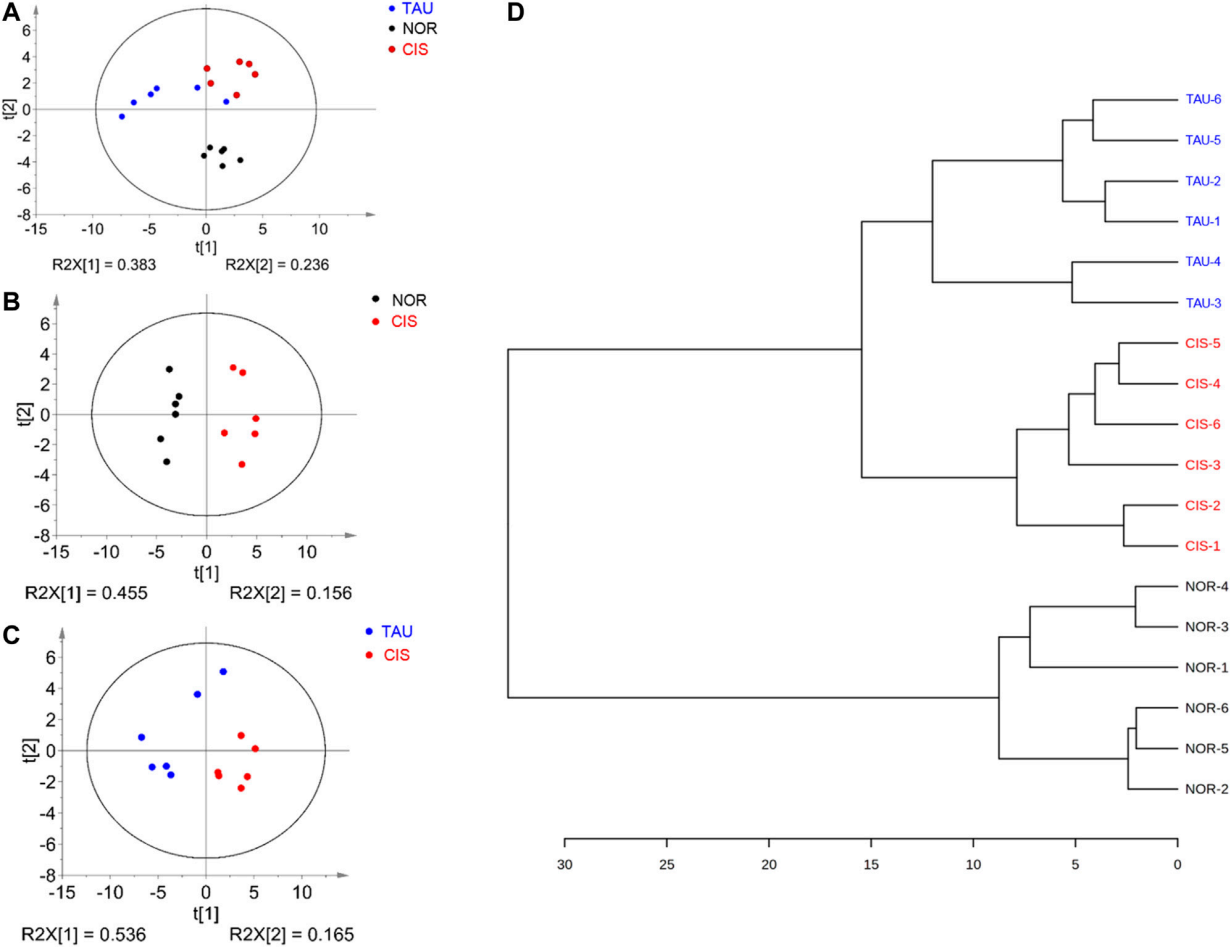
Pattern recognition analysis for the 1D ^1^H NMR spectra recorded on aqueous extracts derived from the three groups of C2C12 myoblasts. **(A)** PCA score plot of the three groups of cells. **(B, C)** PCA score plots for CIS vs. NOR **(B)** and TAU vs. CIS **(C)**. The ellipses indicate the 95% confidence limits. **(D)** Hierarchical cluster analysis for the three groups of cells.

To avoid the potential perturbation of supplemented taurine to metabolic profiling of C2C12 myoblasts, we excluded taurine-related NMR data from the multivariate data analysis. Unsupervised PCA models were constructed with the first two principal components (pc1 and pc2) to comprehensively compare metabolic profiles among the three groups of cells (Figure 5). The PCA score plot shows that the three groups could be metabolically distinguished basically (Figure 5A). Obviously, the CIS group was metabolically separated from the NOR group, indicating that cisplatin treatment profoundly changed the metabolic profile of myoblasts (Figures 5A,B). Furthermore, the TAU group displayed a metabolic profile distinctly distinguished from the CIS group, reflecting the significant effect of taurine supplementation on the cisplatin-changed metabolic profile of the cells (Figure 5C). In addition, the TAU group was metabolically distinct from the NOR group, implying that taurine supplementation could not fully restore the cisplatin-changed metabolic profile to the normal one. We also conducted the HCA to confirm the metabolic separations among the three groups obtained from the PCA (Figure 5D). As expected, the NOR, CIS, and TAU groups of cells were classified as three distinct clusters, well supporting the PCA grouping results.

**FIGURE 6:**
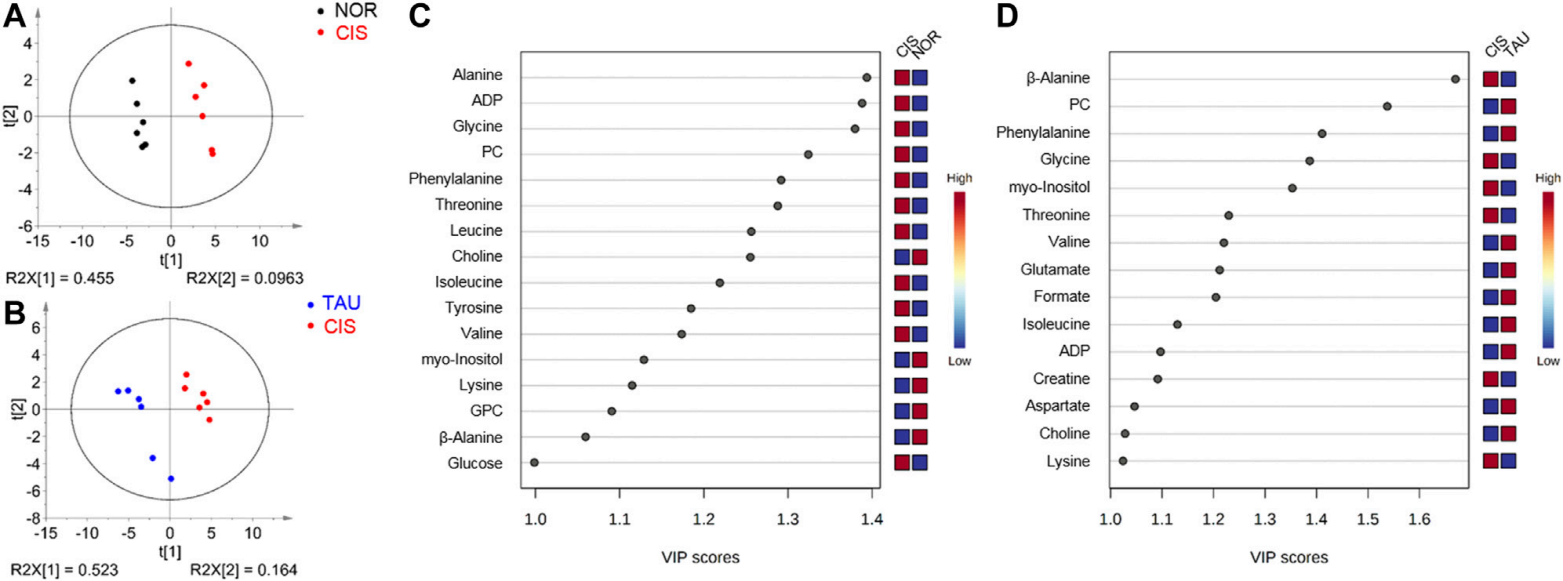
PLS-DA analysis for identifying significant metabolites primarily responsible for the discrimination of metabolic profiles among the NOR, CIS, and TAU groups of C2C12 myoblasts. **(A, B)** PLS-DA score plots for CIS vs. NOR **(A)** and TAU vs. CIS **(B)**. The ellipses indicate the 95% confidence limits. **(C, D)** VIP score-ranking plots of significant metabolites identified from the PLS-DA models of CIS vs. NOR **(C)** and TAU vs. CIS **(D)**. Significant metabolites were identified with VIP >1.

To obtain better metabolic separations between the three groups of cells, we established PLS-DA models based on the NMR spectral data and the grouping information. The PLS-DA score plots illustrate excellent metabolic distinctions between the CIS and NOR groups and between the TAU and CIS groups (Figures 6A,B). Furthermore, the cross-validation plots were obtained by response permutation tests using the first two components (*n* = 200), which exhibit the good robustness of the PLS-DA models (Supplementary Figure S5).

### Identification of Characteristic Metabolites

The PLS-DA models were applied to identify significant metabolites that were primarily responsible for the metabolic separations. Totally, 16 and 15 significant metabolites were identified from the PLS-DA models of the CIS vs. NOR and TAU vs. CIS groups, respectively (Figures 6C,D). Significantly, 11 significant metabolites were shared by the pairwise comparisons of CIS vs. NOR and TAU vs. CIS. Interestingly, eight metabolites (lysine, valine, isoleucine, β-alanine, phenylalanine, myo-inositol, ADP, and PC) were increased in the CIS myoblasts relative to the NOR myoblasts but decreased in the TAU myoblasts relative to the CIS myoblasts.

To quantitatively compare metabolite levels among the three groups of myoblasts, we calculated absolute concentrations of the 29 identified metabolites (except taurine) based on their peak integrals relative to the peak integral of TSP as an inner reference molecule with a known concentration. Accordingly, differential metabolites were identified from univariate analyses of metabolite levels for NOR, CIS, and TAU myoblasts (Table 2). The identified differential metabolites were almost consistent with the significant metabolites identified from the PLS-DA models.

**TABLE 2:** Multiple comparisons of metabolite levels among the NOR, CIS, and TAU groups of C2C12 myoblasts based on relative NMR integrals.

Metabolite	Mean ± SD			Multiple comparisons		One-way ANOVA	
	NOR	CIS	TAU	CIS vs NOR	TAU vs CIS	*F*	*P*
Leucine	0.175 ± 0.005	0.195 ± 0.012	0.169 ± 0.018	↑	↓	6.582	0.009
Isoleucine	0.089 ± 0.004	0.101 ± 0.007	0.088 ± 0.010	↑	↓	4.892	0.023
Valine	0.110 ± 0.005	0.118 ± 0.008	0.105 ± 0.014	NS	NS	3.086	0.075
Alanine	0.404 ± 0.014	0.562 ± 0.022	0.435 ± 0.054	↑↑↑	↓↓	35.113	<0.001
Lysine	0.112 ± 0.005	0.098 ± 0.008	0.113 ± 0.007	↓↓	↑↑↑	55.603	<0.001
Acetate	0.484 ± 0.199	0.496 ± 0.201	0.393 ± 0.179	NS	NS	0.509	0.611
Glutamate	0.409 ± 0.033	0.425 ± 0.021	0.381 ± 0.034	NS	NS	3.290	0.065
Glutamine	0.276 ± 0.015	0.251 ± 0.011	0.276 ± 0.015	↓	↑↑↑	38.690	<0.001
Asparate	0.022 ± 0.002	0.021 ± 0.004	0.022 ± 0.002	NS	NS	0.011	0.989
Glutathione	0.131 ± 0.006	0.133 ± 0.013	0.119 ± 0.023	NS	NS	1.485	0.258
β-Alanine	0.122 ± 0.008	0.106 ± 0.008	0.120 ± 0.005	↓↓	↑↑↑	362.700	<0.001
Choline	0.055 ± 0.004	0.040 ± 0.004	0.051 ± 0.022	↓↓↓	NS	1.958	0.176
PC	0.249 ± 0.035	0.450 ± 0.028	0.294 ± 0.023	↑↑↑	↓↓↓	210.673	<0.001
GPC	0.660 ± 0.137	0.430 ± 0.066	0.393 ± 0.080	↓↓	NS	12.695	0.001
Glycine	0.576 ± 0.019	0.827 ± 0.034	0.561 ± 0.040	↑↑↑	↓↓↓	131.264	<0.001
Creatine	0.454 ± 0.118	0.434 ± 0.068	0.284 ± 0.075	NS	↓	6.427	0.010
Phosphocreatine	0.456 ± 0.060	0.564 ± 0.069	0.452 ± 0.052	↑	↓	6.538	0.009
Myo-inositol	0.539 ± 0.053	0.429 ± 0.052	0.527 ± 0.076	↓	↑	31.988	<0.001
Lactate	0.266 ± 0.036	0.279 ± 0.032	0.233 ± 0.102	NS	NS	0.797	0.469
Threonine	0.067 ± 0.007	0.087 ± 0.003	0.061 ± 0.009	↑↑↑	↓↓↓	22.860	<0.001
Glucose	0.039 ± 0.014	0.072 ± 0.024	0.053 ± 0.020	↑	NS	4.101	0.038
ADP	0.007 ± 0.001	0.026 ± 0.002	0.027 ± 0.004	↑	NS	121.181	<0.001
ATP	0.081 ± 0.006	0.084 ± 0.003	0.071 ± 0.007	NS	↓↓	10.158	0.002
Histidine	0.006 ± 0.001	0.006 ± 0.001	0.005 ± 0.001	NS	NS	3.668	0.050
Tyrosine	0.049 ± 0.003	0.053 ± 0.003	0.046 ± 0.005	NS	↓↓	6.016	0.012
Phenylalanine	0.037 ± 0.001	0.041 ± 0.003	0.037 ± 0.004	↑	NS	3.686	0.050
GTP	0.032 ± 0.002	0.034 ± 0.002	0.032 ± 0.004	NS	NS	0.755	0.487
Formate	0.008 ± 0.001	0.009 ± 0.001	0.009 ± 0.002	NS	NS	0.904	0.426
NAD^+^	0.016 ± 0.002	0.014 ± 0.001	0.011 ± 0.002	↓	NS	11.787	0.001

Note: Statistical significances were determined by one-way ANOVA followed by Tukey’s multiple comparison test and represented by *p* values: NS, *p* > 0.05; ↓/↑, *p* < 0.05; ↓↓/↑↑, *p* < 0.01; ↓↓↓/↑↑↑, *p* < 0.001. Differential metabolites were identified with *p* < 0.05. The upward arrow and the downward arrow denote that the difference between A and B is positive (A is increased compared to B) and negative (A is decreased compared to B), respectively.

**FIGURE 7:**
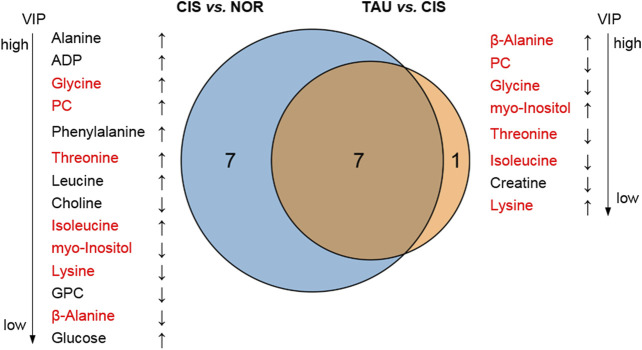
Venn diagrams of characteristic metabolites identified from pairwise comparisons of CIS vs. NOR and TAU vs. CIS. Characteristic metabolites were determined by a combination of the significant metabolites identified from the PLS-DAs (VIP >1) and differential metabolites identified from the univariate analyses (*p* < 0.05). The upward arrow and downward arrow denote that the difference between A and B is positive (A is increased compared to **(B)**) and negative (A is decreased compared to **(B)**), respectively. The metabolites in red font refer to shared characteristic metabolites in the two comparisons.

The combination of the significant metabolites and differential metabolites gave characteristic metabolites for the pairwise comparisons between the three groups of myoblasts. We thus identified 14 and 8 characteristic metabolites for the comparisons of CIS vs. NOR and TAU vs. CIS, respectively (Figure 7). A total of 7 characteristic metabolites were shared by the pairwise comparisons between CIS and NOR myoblasts, and between TAU and CIS myoblasts. Interestingly, 4 of the 7 shared characteristic metabolites (threonine, isoleucine, glycine, and PC) were increased in CIS myoblasts relative to NOR myoblasts but consistently decreased in TAU myoblasts relative to CIS myoblasts. Another 3 shared characteristic metabolites (β-alanine, lysine, and myo-inositol) were declined in CIS myoblasts relative to NOR myoblasts but consistently elevated in TAU myoblasts relative to CIS myoblasts (Figure 7).

Furthermore, to explore potential relationships between the oxidative stress state/cell viability and cellular metabolic profile impaired by cisplatin treatment, we analyzed correlations of ROS and MDA levels and cell viabilities with intracellular levels of the 14 characteristic metabolites in CIS myoblasts, which were identified from the comparison of CIS vs. NOR as described above (Supplementary Figure S6). The ROS/MDA levels showed positive correlations with 9 metabolites (glycine, PC, alanine, threonine, ADP, phenylalanine, leucine, isoleucine, and glucose) and negative correlations with 5 metabolites (lysine excluding for ROS-related correlation, choline, GPC, β-alanine, and myo-inositol). Interestingly, the cell viability–related correlations showed exactly opposite signs to the MDA-related correlations (Supplementary Figure S6) probably due to the abnormal oxidative stress leading to decreased cell viability.

### Identification of Significantly Altered Metabolic Pathways

**FIGURE 8:**
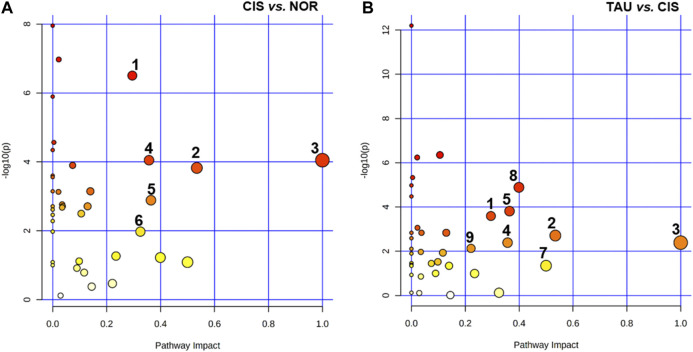
Significantly altered metabolic pathways identified from pairwise comparisons of CIS vs. NOR and TAU vs. CIS. The significant pathways were identified with pathway impact values >0.2 and *p* values <0.05, using the Pathway Analysis module provided by MetaboAnalyst 4.0 webserver. Numbers in the panels represent significantly altered metabolic pathways: (1) glycine, serine, and threonine metabolism; (2) alanine, aspartate, and glutamate metabolism; (3) phenylalanine, tyrosine, and tryptophan biosynthesis; (4) phenylalanine metabolism; (5) glutathione metabolism; (6) starch and sucrose metabolism; (7) D-glutamine and D-glutamate metabolism; (8) β-alanine metabolism; and (9) histidine metabolism.

We performed metabolic pathway analyses to identify significantly altered metabolic pathways (defined as significant pathways) with two criteria of pathway impact value >0.2 and *p* value <0.05. A total of six and eight significant pathways were identified from the pairwise comparisons of CIS vs. NOR and TAU vs. CIS, respectively (Figure 8). The two comparisons shared five significant pathways: (1) glycine, serine, and threonine metabolism; (2) alanine, aspartate, and glutamate metabolism; (3) phenylalanine, tyrosine, and tryptophan biosynthesis; (4) phenylalanine metabolism; and (5) glutathione metabolism.

Compared with NOR myoblasts, CIS myoblasts showed one extra significant pathway: (6) starch and sucrose metabolism. Compared to CIS myoblasts, TAU myoblasts displayed three extra significant pathways: (7) D-glutamine and D-glutamate metabolism, (8) β-alanine metabolism, and (9) histidine metabolism.

**FIGURE 9:**
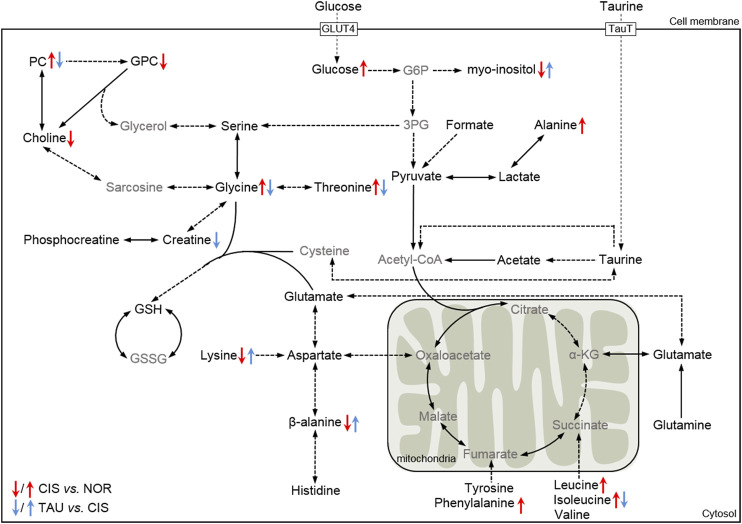
Schematic representation of significantly altered metabolic pathways of C2C12 myoblasts under cisplatin treatment and taurine supplementation based on the KEGG database. Dotted arrow indicates multiple biochemical reactions; solid arrow denotes single biochemical reaction. The metabolites in black/gray font refer to detected/undetected metabolites. Abbreviations: GLUT4, glucose transporter 4; TauT, taurine transporter; G6P, glucose-6-phosphate; 3-PG, 3-phosphoglycerate; α-KG, α-ketoglutarate; GSH, reduced glutathione; GSSG; oxidized glutathione.

To systemically visualize the significant changes in intracellular metabolite levels of CIS myoblasts relative to NOR myoblasts, and TAU myoblasts relative to CIS myoblasts, we projected these characteristic metabolites onto a metabolic map based on the Kyoto Encyclopedia of Genes and Genomes (KEGG) database (Figure 9). In general, the changing trends of some metabolite levels in CIS myoblasts relative to NOR myoblasts were opposite to those in TAU myoblasts relative to CIS myoblasts, indicating that taurine supplementation could partially reverse the metabolic changes impaired by cisplatin treatment.

## Discussion

We have revealed the protective effects of taurine supplementation on C2C12 myoblasts impaired by cisplatin treatment and clarified the underlying molecular mechanisms. Compared with the NOR myoblasts, the CIS myoblasts showed aberrant intracellular ROS accumulation and suppressed cellular proliferation. Furthermore, the CIS myoblasts exhibited impaired myotube differentiation ability and decreased expressions of MyoD1, myogenin, and MHC as well as a decreased ratio of p-AKT1 (ser473)/AKT1 compared to the NOR myoblasts. To some extent, taurine supplementation could protect myoblasts against cisplatin-induced cell viability decrease, facilitate cellular ROS clearance, and, most importantly, preserved the expression of MyoD1, myogenin, and MHC; increased the ratio of p-AKT1 (ser473)/AKT1; and maintained the myotube differentiation ability of myoblasts. Furthermore, the CIS myoblasts showed that cisplatin treatment increased the levels of BCAA (isoleucine), threonine, glycine, and PC, and decreased the levels of β-alanine, lysine, and myo-inositol compared to the NOR myoblasts. Note that the cisplatin-induced changing trends of these metabolites were effectively reversed in taurine-supplemented myoblasts. Our results reveal that taurine exerts the protective effects for improving the proliferation and myotube differentiation ability of C2C12 myoblasts impaired by cisplatin by alleviating cellular catabolism, facilitating GSH biosynthesis, improving glucose utilization and TCA cycle anaplerosis, and stabilizing lipid membranes.

Skeletal muscle is one of the three muscle tissues in the human body, which cooperates with bone, nerve, and blood vascular tissue and thus coordinates and supports body movement (Bottinelli and Reggiani, 2000; Frontera and Ochala, 2015). It is worth noting that high-intensity physical exercise of athletes arouses systemic alteration in several biochemical states of skeletal muscle cells, including metabolic changes, ROS production, and consequent oxidative stress–induced muscle injury (Cheng et al., 2020). Similarly, about 70%–80% of cancer patients experience muscle injury during chemotherapy, resulting in muscular functional impairments, sarcopenia, or even cachexia (Kodera, 2015; Chen et al., 2016; Kakinuma et al., 2018; Matsuura et al., 2020). Potentially, chemotherapeutic drugs can induce ROS, resulting in muscle injury (Kodera, 2015; Matsuura et al., 2020). For example, cisplatin is widely used to combat multiple types of cancers, which is accompanied by side effects of inducing oxidative stress in muscle cells (Sakai et al., 2014; Conte et al., 2020). It is expected that antioxidative interventions could be applied to attenuate oxidative stress–induced muscle injury. Typically in athletes, taurine-containing sports beverages have long been used for accelerating postexercise muscle recovery (Alford et al., 2001; van den Eynde et al., 2008). Furthermore, taurine has been acknowledged as an alternative candidate for relieving muscular toxicity associated with oxidative stress induced by chemotherapeutic agents. Previous studies have documented the antioxidant functions of taurine in myotubes and myofibers rather than myoblasts (Stacchiotti et al., 2014; Seidel et al., 2019; Ma et al., 2021). It remains unclear how taurine plays a crucial role in the maintenance of muscular homeostasis. In this study, we focused on the effects of taurine supplementation on myoblasts, which act as myotube precursors and hold a pivotal role in muscle regeneration.

Expectedly, our study showed that cisplatin treatment dramatically increased intracellular ROS in C2C12 myoblasts, as directly manifested by the increased fluorescent intensity of H_2_DCFDA. Excessive free radicals induce lipid peroxidation with elevated level of the end product MDA, which can cause cross-linking and polymerization of proteins, nucleic acids, and other macromolecules. Cisplatin treatment increased MDA in CIS myoblasts, indicating promoted cellular lipid peroxidation. Significantly, taurine supplementation decreased MDA in TAU myoblasts. For the first time, we found that taurine supplementation can preserve the myotube differentiation ability of C2C12 myoblasts and partially restore MyoD1 expression in the cells upon cisplatin treatment.

It is well known that myoblasts, also named myogenic satellite cells, are the primary source of muscle repair and regeneration in damaged myofibers (Shi and Garry, 2006; Dumont et al., 2015; Frontera and Ochala, 2015; Joanisse et al., 2017). In response to muscle damage stimulus, quiescent myoblast cells are capable of re-entering the cell cycle to proliferate and then differentiate to form myotubes (Chargé and Rudnicki, 2004; Baghdadi and Tajbakhsh, 2018). We found that cisplatin treatment resulted in a one-fold decrease in cell viability of C2C12 myoblasts. Moreover, the expression of MyoD1, myogenin, and MHC was dramatically decreased in CIS myoblasts, implying an impaired myotube differentiation ability of the myoblasts. As expected, the reduced proliferation rate and decreased expression of MyoD1, myogenin, and MHC would lead to impeded myotube formation in CIS myotubes, which was manifested by the in vitro experimental observation of remarkably decreased myotube area. Additionally, TAU myoblasts showed slightly increased cell viability and moderately promoted the expression of MyoD1, myogenin, and MHC upon taurine supplementation. The improved myotube formation was further confirmed by the in vitro experimental observation of increased myotube area in the TAU myoblasts relative to the CIS myoblasts. Thus, our work further demonstrates the protective effects of taurine treatment on cisplatin-impaired myoblasts. Given that taurine did not exert significant protective effects on C2C12 myoblasts when added at the same time point of cisplatin treatment (data not shown), we here adopted a pre-supplementation strategy in which taurine was added in DMEM for 12 h before cisplatin treatment. Prospectively, we suggest that taurine should be pre-supplemented when applied to alleviate muscle toxicities of chemotherapeutic drugs in cancer patients.

Previous studies have shown that cisplatin can induce cellular autophagy, thus promoting cellular catabolism (Sakai et al., 2014; Sakai et al., 2020). In this study, CIS myoblasts showed increased intracellular levels of several amino acids, and the increased levels of BCAAs (leucine and isoleucine), glycine, alanine, threonine, phenylalanine, PC, ADP, and glucose were positively correlated with the increased ROS/MDA levels. Furthermore, CIS myoblasts also displayed decreased intracellular levels of myo-inositol, choline, lysine, β-alanine, and GPC, which were negatively correlated with the ROS/MDA levels.

### Taurine Supplementation Promotes GSH Biosynthesis and Facilitates ROS Clearance

Expectedly, the increased intracellular ROS level in CIS myoblasts would enhance GSH consumption, leading to decreased GSH levels. Interestingly, CIS myoblasts did not show statistically significantly different GSH levels compared to NOR myoblasts. As it is known, three metabolites (glycine, cysteine, and glutamate) act as precursors for glutathione biosynthesis. The increased levels of threonine and glycine in CIS myoblasts relative to NOR myoblasts did not significantly change the GSH level potentially due to insufficient cysteine, thereby being not able to promote the clearance of cisplatin-induced ROS. Profoundly, taurine supplementation dramatically raised the intracellular level of taurine. The supplemented taurine could be converted into cysteine, providing sufficient cysteine as the precursor to glutathione. Thus, glutathione biosynthesis was greatly upregulated in TAU myoblasts, as manifested by the decreased levels of threonine and glycine which were used for glutathione biosynthesis. These results indicate that taurine supplementation greatly promotes glutathione biosynthesis and thereby facilitates the clearance of intracellular ROS.

In this study, we also treated C2C12 myoblasts only with taurine and monitored the metabolites without adding cisplatin. Compared to normal C2C12 myoblasts (NOR), taurine-treated C2C12 myoblasts (NOR–TAU) showed several significantly changed metabolites, including increased GSH (*p* < 0.01), decreased β-alanine (*p* < 0.001), and decreased phosphocreatine (*p* < 0.05). These results suggested that taurine supplementation promoted GSH synthesis even in normal C2C12 myoblasts. Furthermore, the PCA score plot of the four cell groups (NOR, CIS, TAU, and NOR–TAU) exhibits that taurine supplementation shifted the metabolic profile of normal C2C12 cells less than cisplatin treatment (data not showed).

### Taurine Supplementation Improves Cellular Glucose Utilization and TCA Cycle Anaplerosis

As is known, both the glycolysis pathway and TCA cycle are the core energy-producing pathways. The accumulated glucose and decreased myo-inositol levels were indicative of impaired glucose utilization in CIS myoblasts, whereas the accumulated intracellular BCAAs (leucine and isoleucine) and phenylalanine potentially implied activated TCA cycle anaplerosis. Therefore, it seemed that CIS myoblasts could meet the energy demand by upregulating TCA cycle anaplerosis. Significantly, taurine supplementation restored the increased level of isoleucine and decreased level of myo-inositol in myoblasts impaired by cisplatin treatment, thereby improving glucose utilization and TCA cycle anaplerosis.

### Taurine Supplementation Ameliorates Cisplatin-Promoted Cellular Catabolism

Our observation of increased amino acids in CIS myoblasts is in agreement with the documented effects of cisplatin treatment for promoting cellular catabolism. Previous studies have emphasized the important role of organismal alanine metabolism (Derave et al., 2010; Berti Zanella et al., 2017). Upon activation of protein catabolism in the muscle, alanine works as a primary substrate for amino acid transformation and gluconeogenesis (Derave et al., 2010; Berti Zanella et al., 2017). In this study, CIS myoblasts showed a significant upregulated level of alanine and obviously downregulated levels of lysine and β-alanine. As is known, lysine is accumulated once cellular catabolism is promoted, which is preferentially converted into aspartate together with β-alanine for further oxidation in the TCA cycle. The lysine and β-alanine–derived aspartate also can be utilized for glutamate production and thus contribute to the unfulfilled GSH synthesis. Significantly, taurine supplementation restored the cisplatin-decreased levels of lysine and β-alanine, thus ameliorating cellular catabolism promoted by cisplatin treatment.

### Taurine Supplementation Protects the Integrity of Cellular Membranes

As is known, choline is an essential anabolic substrate for synthesizing phospholipids, which is phosphorylated by choline kinase to PC (phosphocholine), which serves as a precursor for phosphatidylcholine synthesis. Phosphatidylcholine is the major phospholipid constituent of cellular membranes, and glycerophospholipid metabolism potentially affects cellular membrane stability (Vance, 2008; Furse and de Kroon, 2015). Under the circumstance that lipid membranes are disrupted by ROS-induced lipid peroxidation, the promoted phosphatidylcholine synthesis is a prerequisite for the maintenance of membrane integrity. Our study detected a significantly increased intracellular level of PC and a remarkably decreased level of choline in CIS myoblasts relative to NOR myoblasts, implying a promoted phosphatidylcholine synthesis for cellular membrane repair. On the contrary, taurine supplementation alleviated lipid peroxidation by promoting ROS clearance, and decreased phosphatidylcholine consumption, as indicated by the decreased level of PC in TAU myoblasts relative to CIS myoblasts.

## Conclusion

We have demonstrated that taurine supplementation protected C2C12 myoblasts against cell viability decrease, facilitated intracellular ROS clearance, and, most importantly, partially preserved the myotube differentiation ability of cisplatin-impaired myoblasts. Cisplatin treatment increases levels of BCAAs (leucine and isoleucine), glycine, and PC, and decreases levels of β-alanine and myo-inositol in C2C12 myoblasts. Significantly, taurine supplementation partially reverses the changing trends of these metabolite levels, primarily by improving cellular catabolism, promoting GSH biosynthesis, improving glucose utilization and TCA cycle anaplerosis, and stabilizing cellular membranes in myoblasts impaired by cisplatin treatment. For the first time, this work revealed the protective effects of taurine supplementation for preserving the myotube differentiation ability of cisplatin-treated C2C12 myoblasts. Our results suggest that nutritional supplementation of taurine may be a promising approach for ameliorating muscle toxicities in cancer patients undergoing chemotherapy.

## Data Availability

The original contributions presented in the study are included in the article/Supplementary Material. Further inquiries can be directed to the corresponding author.

## Glossary

ADP: adenosine diphosphate
BCAAs: branched-chain amino acids
CIS: cisplatin-treated
ETC: electron transport chain
FID: free induction delay
GPC: sn-glycero-3-phosphocholine
GSH: reduced glutathione
H2DCFDA: 2′,7′-dichlorofluorescin diacetate
HCA: hierarchical cluster analysis
HMDB: Human Metabolome Data Base
HSQC: heteronuclear single quantum coherence
KEGG: Kyoto Encyclopedia of Genes and Genomes
MDA: malondialdehyde
MTS: 3-(4,5-dimethylthiazol-2-yl)-5-(3-carboxymethoxyphenyl)-2-(4-sulfophenyl)-2H-tetrazolium, inner salt
MyoD1: myoblast determination protein 1
NAD+: nicotinamide adenine dinucleotide
NOR: normal control
NOR-TAU cells: taurine-supplemented normal cells
PC: O-phosphocholine
PCA: principal component analysis
PLS-DA: partial least-squares discriminant analysis
ROS: reactive oxygen species
TAU: cisplatin-treated taurine-supplemented
TauT: taurine transporter
TOCSY: total correlation spectroscopy
TSP: sodium 3-(trimethylsilyl) propionate-2,2,3,3-d4
VIP: variable importance in projection

## References

[B1] Alford C. Cox H. Wescott R. (2001). The Effects of Red Bull Energy Drink on Human Performance and Mood. Amino Acids 21 (2), 139–150. 10.1007/s007260170021 11665810

[B2] Asakura A. Fujisawa-Sehara A. Komiya T. Nabeshima Y. Nabeshima Y. (1993). MyoD and Myogenin Act on the Chicken Myosin Light-Chain 1 Gene as Distinct Transcriptional Factors. Mol. Cel. Biol. 13 (11), 7153–7162. 10.1128/mcb.13.11.7153 PMC364776 8413304

[B3] Baghdadi M. B. Tajbakhsh S. (2018). Regulation and Phylogeny of Skeletal Muscle Regeneration. Develop. Biol. 433 (2), 200–209. 10.1016/j.ydbio.2017.07.026 28811217

[B4] Beckonert O. Keun H. C. Ebbels T. M. D. Bundy J. Holmes E. Lindon J. C. (2007). Metabolic Profiling, Metabolomic and Metabonomic Procedures for NMR Spectroscopy of Urine, Plasma, Serum and Tissue Extracts. Nat. Protoc. 2 (11), 2692–2703. 10.1038/nprot.2007.376 18007604

[B5] Berti Zanella P. Donner Alves F. Guerini de Souza C. (2017). Effects of Beta-Alanine Supplementation on Performance and Muscle Fatigue in Athletes and Non-athletes of Different Sports: a Systematic Review. J. Sports Med. Phys. Fitness 57 (9), 1132–1141. 10.23736/s0022-4707.16.06582-8 27377257

[B6] Bottinelli R. Reggiani C. (2000). Human Skeletal Muscle Fibres: Molecular and Functional Diversity. Prog. Biophys. Mol. Biol. 73 (2-4), 195–262. 10.1016/s0079-6107(00)00006-7 10958931

[B7] Buckingham M. Rigby P. W. J. (2014). Gene Regulatory Networks and Transcriptional Mechanisms that Control Myogenesis. Develop. Cel 28 (3), 225–238. 10.1016/j.devcel.2013.12.020 24525185

[B8] Chargé S. B. P. Rudnicki M. A. (2004). Cellular and Molecular Regulation of Muscle Regeneration. Physiol. Rev. 84 (1), 209–238. 10.1152/physrev.00019.2003 14715915

[B9] Chen L. Han X. Hu Z. Chen L. (2019). The PVT1/miR-216b/Beclin-1 Regulates Cisplatin Sensitivity of NSCLC Cells via Modulating Autophagy and Apoptosis. Cancer Chemother. Pharmacol. 83 (5), 921–931. 10.1007/s00280-019-03808-3 30859368

[B10] Chen M.-C. Hsu W.-L. Hwang P.-A. Chen Y.-L. Chou T.-C. (2016). Combined Administration of Fucoidan Ameliorates Tumor and Chemotherapy-Induced Skeletal Muscle Atrophy in Bladder Cancer-Bearing Mice. Oncotarget 7 (32), 51608–51618. 10.18632/oncotarget.9958 27323407 PMC5239500

[B11] Cheng A. J. Jude B. Lanner J. T. (2020). Intramuscular Mechanisms of Overtraining. Redox Biol. 35, 101480. 10.1016/j.redox.2020.101480 32179050 PMC7284919

[B12] Conte E. Bresciani E. Rizzi L. Cappellari O. De Luca A. Torsello A. (2020). Cisplatin-Induced Skeletal Muscle Dysfunction: Mechanisms and Counteracting Therapeutic Strategies. Int J Mol Sci. 21 (4), 1242. 10.3390/ijms21041242 PMC7072891 32069876

[B13] Cui P. Huang C. Guo J. Wang Q. Liu Z. Zhuo H. (2019a). Metabolic Profiling of Tumors, Sera, and Skeletal Muscles from an Orthotopic Murine Model of Gastric Cancer Associated-Cachexia. J. Proteome Res. 18 (4), 1880–1892. 10.1021/acs.jproteome.9b00088 30888184

[B14] Cui P. Shao W. Huang C. Wu C.-J. Jiang B. Lin D. (2019b). Metabolic Derangements of Skeletal Muscle from a Murine Model of Glioma Cachexia. Skeletal Muscle 9 (1), 3. 10.1186/s13395-018-0188-4 30635036 PMC6330447

[B15] Derave W. Everaert I. Beeckman S. Baguet A. (2010). Muscle Carnosine Metabolism and β-Alanine Supplementation in Relation to Exercise and Training. Sports Med. 40 (3), 247–263. 10.2165/11530310-000000000-00000 20199122

[B16] Dumont N. A. Bentzinger C. F. Sincennes M.-C. Rudnicki M. A. (2015). Satellite Cells and Skeletal Muscle Regeneration. Compr. Physiol. 5 (3), 1027–1059. 10.1002/cphy.c140068 26140708

[B17] Franconi F. Loizzo A. Ghirlanda G. Seghieri G. (2006). Taurine Supplementation and Diabetes Mellitus. Curr. Opin. Clin. Nutr. Metab. Care 9 (1), 32–36. 10.1097/01.mco.0000196141.65362.46 16444816

[B18] Froger N. Sahel J.-A. Picaud S. (2014). “Taurine Deficiency and the Eye,” in Handbook of Nutrition, Diet and the Eye. Editor Preedy V. R. (San Diego: Academic Press), 505–513. 10.1016/b978-0-12-401717-7.00051-4

[B19] Frontera W. R. Ochala J. (2015). Skeletal Muscle: a Brief Review of Structure and Function. Calcif Tissue Int. 96 (3), 183–195. 10.1007/s00223-014-9915-y 25294644

[B20] Furse S. de Kroon A. I. P. M. (2015). Phosphatidylcholine’s Functions beyond that of a Membrane Brick. Mol. Membr. Biol. 32 (4), 117–119. 10.3109/09687688.2015.1066894 26306852

[B21] Ganassi M. Badodi S. Wanders K. Zammit P. S. Hughes S. M. (2020). Myogenin Is an Essential Regulator of Adult Myofibre Growth and Muscle Stem Cell Homeostasis. Elife 9, e60445. 10.7554/eLife.60445 33001028 PMC7599067

[B22] Han X. Chesney R. W. (2006). Mechanisms of Regulation of Taurine Transporter Activity. Adv. Exp. Med. Biol. 583, 79–90. 10.1007/978-0-387-33504-9_8 17153591

[B23] Han X. Patters A. B. Jones D. P. Zelikovic I. Chesney R. W. (2006). The Taurine Transporter: Mechanisms of Regulation. Acta Physiol. 187 (1-2), 61–73. 10.1111/j.1748-1716.2006.01573.x 16734743

[B24] Huxtable R. J. Lippincott S. E. (1982). Diet and Biosynthesis as Sources of Taurine in the Mouse. J. Nutr. 112 (5), 1003–1010. 10.1093/jn/112.5.1003 7077412

[B25] Joanisse S. Nederveen J. P. Snijders T. McKay B. R. Parise G. (2017). Skeletal Muscle Regeneration, Repair and Remodelling in Aging: The Importance of Muscle Stem Cells and Vascularization. Gerontology 63 (1), 91–100. 10.1159/000450922 27760421

[B26] Kakinuma K. Tsuruoka H. Morikawa K. Furuya N. Inoue T. Miyazawa T. (2018). Differences in Skeletal Muscle Loss Caused by Cytotoxic Chemotherapy and Molecular Targeted Therapy in Patients with Advanced Non-small Cell Lung Cancer. Thorac. Cancer 9 (1), 99–104. 10.1111/1759-7714.12545 29067769 PMC5754304

[B27] Kodera Y. (2015). More Than 6 Months of Postoperative Adjuvant Chemotherapy Results in Loss of Skeletal Muscle: a Challenge to the Current Standard of Care. Gastric Cancer 18 (2), 203–204. 10.1007/s10120-014-0381-z 24820695

[B28] Lejri I. Agapouda A. Grimm A. Eckert A. (2019). Mitochondria- and Oxidative Stress-Targeting Substances in Cognitive Decline-Related Disorders: From Molecular Mechanisms to Clinical Evidence. Oxidative Med. Cell Longevity 2019, 1–26. 10.1155/2019/9695412 PMC6535827 31214285

[B29] Leon R. Wu H. Jin Y. Wei J. Buddhala C. Prentice H. (2009). Protective Function of Taurine in Glutamate-Induced Apoptosis in Cultured Neurons. J. Neurosci. Res. 87 (5), 1185–1194. 10.1002/jnr.21926 18951478

[B30] Liu L. Fan J. Ai G. Liu J. Luo N. Li C. (2019). Berberine in Combination with Cisplatin Induces Necroptosis and Apoptosis in Ovarian Cancer Cells. Biol. Res. 52 (1), 37. 10.1186/s40659-019-0243-6 31319879 PMC6637630

[B31] Liu Z. Huang C. Liu Y. Lin D. Zhao Y. (2018). NMR-based Metabolomic Analysis of the Effects of Alanyl-Glutamine Supplementation on C2C12 Myoblasts Injured by Energy Deprivation. RSC Adv. 8 (29), 16114–16125. 10.1039/c8ra00819a PMC9080260 35542200

[B32] Lyublinskaya O. G. Ivanova J. S. Pugovkina N. A. Kozhukharova I. V. Kovaleva Z. V. Shatrova A. N. (2017). Redox Environment in Stem and Differentiated Cells: A Quantitative Approach. Redox Biol. 12, 758–769. 10.1016/j.redox.2017.04.016 28426982 PMC5393314

[B33] Ma Y. Maruta H. Sun B. Wang C. Isono C. Yamashita H. (2021). Effects of Long-Term Taurine Supplementation on Age-Related Changes in Skeletal Muscle Function of Sprague-Dawley Rats. Amino Acids 53, 159–170. 10.1007/s00726-020-02934-0 33398526

[B34] Magherini F. Fiaschi T. Marzocchini R. Mannelli M. Gamberi T. Modesti P. A. (2019). Oxidative Stress in Exercise Training: the Involvement of Inflammation and Peripheral Signals. Free Radic. Res. 53 (11-12), 1155–1165. 10.1080/10715762.2019.1697438 31762356

[B35] Matsuura N. Motoori M. Fujitani K. Nishizawa Y. Komatsu H. Miyazaki Y. (2020). Correlation between Skeletal Muscle Mass and Adverse Events of Neoadjuvant Chemotherapy in Patients with Gastric Cancer. Oncology 98 (1), 29–34. 10.1159/000502613 31509833

[B36] Matsuzaki Y. Miyazaki T. Miyakawa S. Bouscarel B. Ikegami T. Tanaka N. (2002). Decreased Taurine Concentration in Skeletal Muscles after Exercise for Various Durations. Med. Sci. Sports Exerc. 34 (5), 793–797. 10.1097/00005768-200205000-00011 11984297

[B37] Militante J. D. Lombardini J. B. (2002). Taurine: Evidence of Physiological Function in the Retina. Nutr. Neurosci. 5 (2), 75–90. 10.1080/10284150290018991 12000086

[B38] Moon P.-D. Kim M.-H. Lim H.-S. Oh H.-A. Nam S.-Y. Han N.-R. (2015). Taurine, a Major Amino Acid of Oyster, Enhances Linear Bone Growth in a Mouse Model of Protein Malnutrition. Biofactors 41 (3), 190–197. 10.1002/biof.1213 25963419

[B39] Oyewole A. O. Birch‐Machin M. A. (2015). Mitochondria‐targeted Antioxidants. FASEB J. 29 (12), 4766–4771. 10.1096/fj.15-275404 26253366

[B40] Sakai H. Ikeno Y. Tsukimura Y. Inomata M. Suzuki Y. Kon R. (2020). Upregulation of Ubiquitinated Proteins and Their Degradation Pathway in Muscle Atrophy Induced by Cisplatin in Mice. Toxicol. Appl. Pharmacol. 403, 115165. 10.1016/j.taap.2020.115165 32738330

[B41] Sakai H. Sagara A. Arakawa K. Sugiyama R. Hirosaki A. Takase K. (2014). Mechanisms of Cisplatin-Induced Muscle Atrophy. Toxicol. Appl. Pharmacol. 278 (2), 190–199. 10.1016/j.taap.2014.05.001 24823295

[B42] Schaffer S. W. Azuma J. Mozaffari M. (2009). Role of Antioxidant Activity of Taurine in diabetesThis Article Is One of a Selection of Papers from the NATO Advanced Research Workshop on Translational Knowledge for Heart Health (Published in Part 1 of a 2-part Special Issue). Can. J. Physiol. Pharmacol. 87 (2), 91–99. 10.1139/y08-110 19234572

[B43] Schaffer S. W. Ju Jong C. Kc R. Azuma J. (2010). Physiological Roles of Taurine in Heart and Muscle. J. Biomed. Sci. 17 (Suppl. 1Suppl 1), S2. 10.1186/1423-0127-17-s1-s2 20804594 PMC2994395

[B44] Seidel U. Huebbe P. Rimbach G. (2019). Taurine: A Regulator of Cellular Redox Homeostasis and Skeletal Muscle Function. Mol. Nutr. Food Res. 63 (16), e1800569. 10.1002/mnfr.201800569 30211983

[B45] Shi X. Garry D. J. (2006). Muscle Stem Cells in Development, Regeneration, and Disease. Genes Dev. 20 (13), 1692–1708. 10.1101/gad.1419406 16818602

[B46] Spriet L. L. Whitfield J. (2015). Taurine and Skeletal Muscle Function. Curr. Opin. Clin. Nutr. Metab. Care 18 (1), 96–101. 10.1097/mco.0000000000000135 25415270

[B47] Stacchiotti A. Rovetta F. Ferroni M. Corsetti G. Lavazza A. Sberveglieri G. (2014). Taurine Rescues Cisplatin-Induced Muscle Atrophy In Vitro: a Morphological Study. Oxid Med. Cell Longev 2014, 1–11. 10.1155/2014/840951 PMC4053152 24955211

[B48] Steinbacher P. Eckl P. (2015). Impact of Oxidative Stress on Exercising Skeletal Muscle. Biomolecules 5 (2), 356–377. 10.3390/biom5020356 25866921 PMC4496677

[B49] Thirupathi A. Freitas S. Sorato H. R. Pedroso G. S. Effting P. S. Damiani A. P. (2018). Modulatory Effects of Taurine on Metabolic and Oxidative Stress Parameters in a Mice Model of Muscle Overuse. Nutrition 54, 158–164. 10.1016/j.nut.2018.03.058 29982143

[B50] Thirupathi A. Pinho R. A. Baker J. S. István B. Gu Y. (2020). Taurine Reverses Oxidative Damages and Restores the Muscle Function in Overuse of Exercised Muscle. Front. Physiol. 11, 582449. 10.3389/fphys.2020.582449 33192592 PMC7649292

[B51] van den Eynde F. van Baelen P. C. Portzky M. Audenaert K. (2008). [The Effects of Energy Drinks on Cognitive Performance]. Tijdschr Psychiatr. 50 (5), 273–281. 18470842

[B52] Vance D. E. (2008). Role of Phosphatidylcholine Biosynthesis in the Regulation of Lipoprotein Homeostasis. Curr. Opin. Lipidol. 19 (3), 229–234. 10.1097/MOL.0b013e3282fee935 18460912

[B53] Wang Q. Li X. Wang Q. Xie J. Xie C. Fu X. (2019a). Heat Shock Pretreatment Improves Mesenchymal Stem Cell Viability by Heat Shock Proteins and Autophagy to Prevent Cisplatin-Induced Granulosa Cell Apoptosis. Stem Cel Res Ther 10 (1), 348. 10.1186/s13287-019-1425-4 PMC6880355 31771642

[B54] Wang Z. Liu G. Jiang J. (2019b). Profiling of Apoptosis- and Autophagy-Associated Molecules in Human Lung Cancer A549 Cells in Response to Cisplatin Treatment Using Stable Isotope Labeling with Amino Acids in Cell Culture. Int. J. Oncol. 54 (3), 1071–1085. 10.3892/ijo.2019.4690 30664195

[B55] Wernig A. (2003). Die Regenerationsfähigkeit der Skelettmuskulatur. Therapeutische Umschau 60 (7), 383–389. 10.1024/0040-5930.60.7.383 12956031

[B56] Xu W. Lin D. Huang C. (2017). NMR-based Metabolomic Analysis for the Effects of Creatine Supplementation on Mouse Myoblast Cell Line C2C12. Acta Biochim. Biophys. Sin (Shanghai) 49 (7), 617–627. 10.1093/abbs/gmx043 28475656

[B57] Zammit P. S. (2017). Function of the Myogenic Regulatory Factors Myf5, MyoD, Myogenin and MRF4 in Skeletal Muscle, Satellite Cells and Regenerative Myogenesis. Semin. Cel Develop. Biol. 72, 19–32. 10.1016/j.semcdb.2017.11.011 29127046

[B58] Zorov D. B. Juhaszova M. Sollott S. J. (2014). Mitochondrial Reactive Oxygen Species (ROS) and ROS-Induced ROS Release. Physiol. Rev. 94 (3), 909–950. 10.1152/physrev.00026.2013 24987008 PMC4101632

