# Supplementary material for: Taurine Protects C2C12 Myoblasts From Impaired Cell Proliferation and Myotube Differentiation Under Cisplatin-Induced ROS Exposure
Source: Front Mol Biosci. 2021 May 26;8:685362. doi: 10.3389/fmolb.2021.685362 (PMC8189557; doi:10.3389/fmolb.2021.685362)
Supplement: Supplementary file 1 [file DataSheet1.docx]

Taurine protects C2C12 myoblasts from impaired cell proliferation and myotube differentiation under cisplatin-induced ROS exposure

Lin Zhou^1^, Ruohan Lu^1^, Caihua Huang^2^, Donghai Lin^1,*^

^1^Key Laboratory for Chemical Biology of Fujian Province, MOE Key Laboratory of Spectrochemical Analysis & Instrumentation, College of Chemistry and Chemical Engineering, Xiamen University, Xiamen 361005, China,

^2^Research and Communication Center of Exercise and Health, Xiamen University of Technology, Xiamen 361024, China.

**Corresponding Authors:**

*Donghai Lin: Key Laboratory for Chemical Biology of Fujian Province, MOE Key Laboratory of Spectrochemical Analysis & Instrumentation, College of Chemistry and Chemical Engineering, Xiamen University, Xiamen 361005, China. Tel: +86-592-2186078, Email: dhlin@xmu.edu.cn.

**Supplementary Figure 1**

**
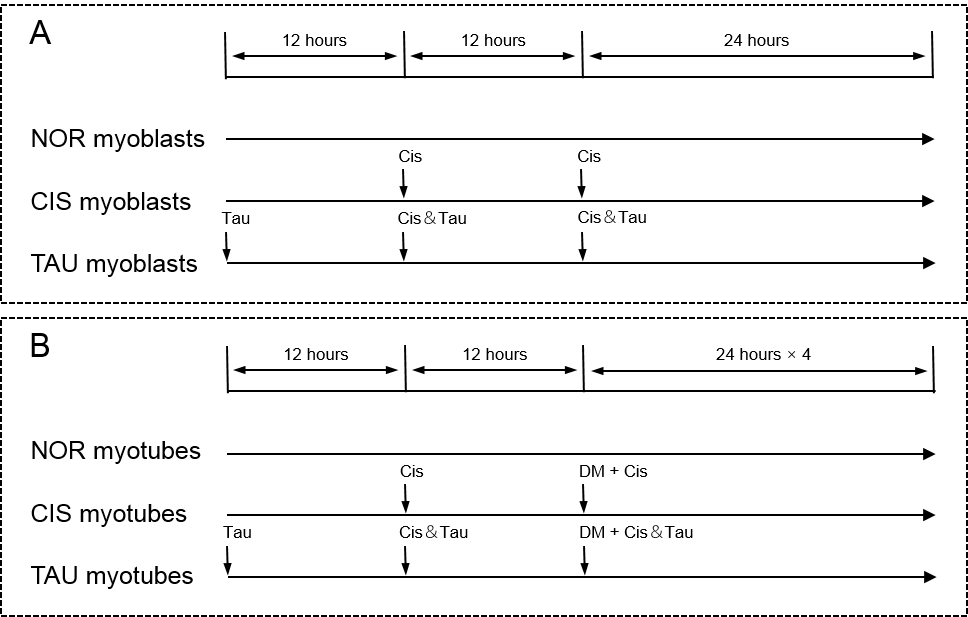
**

**Supplementary Figure.1 Schematic representation of the experimental design. (A)** For *in vitro* myoblast growing experiments Cisplatin was dissolved in DMSO to obtain a 5 mM stock solution and administered to cells at a final concentration of 10 μM for 36 h in the cisplatin-treated (CIS) group. Taurine (5 mM) was added 12 h before cisplatin treatment in the taurine-supplemented (TAU) group. Control experiments were conducted with the equivalent amount of DMSO in the normal control (NOR) group. **(B)** For in vitro myotube differentiation experiments, the C2C12 myoblasts were used within ten generations of culture. When cells reached about 85%-90% confluence after 24 h culture, myotube differentiation was induced by incubation in differentiation medium (DM) for 96 h. Similarly, the same treatment procedures of cisplatin and taurine were carried out in corresponding groups, and the DM was changed every 24 h. (Cis: cisplatin, Tau: taurine)

**Supplementary Figure 2**

**
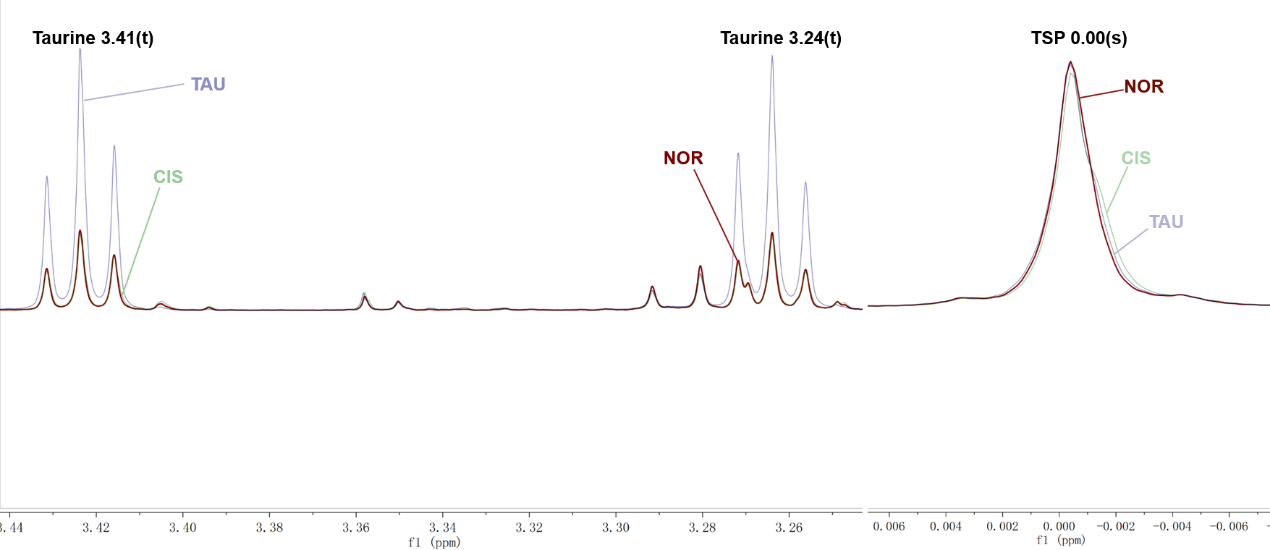
**

**Supplementary Figure 2. Local amplified regions of taurine peaks in typical 1D ^1^H-NMR spectra of aqueous extracts derived from three groups of C2C12 myoblasts recorded on 850 MHz NMR spectrometer.** The TSP peak was used as a chemical shift reference (δ 0.00). Deep red line: spectral region from the NOR group. Light green line: spectral region from the CIS group. Lilac line: spectral region from the TAU group (s, singlet; t, triplet).

**Supplementary Figure 3**


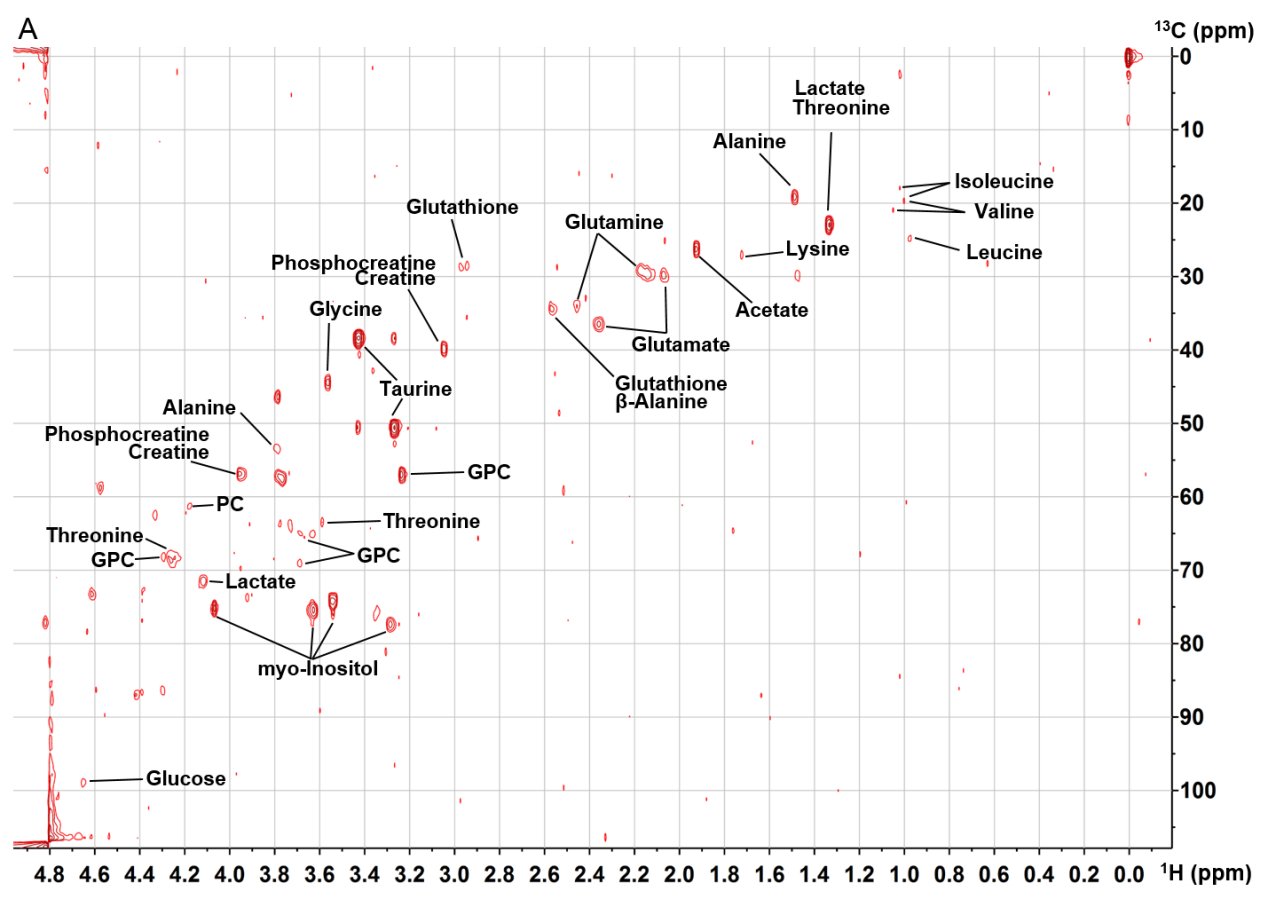

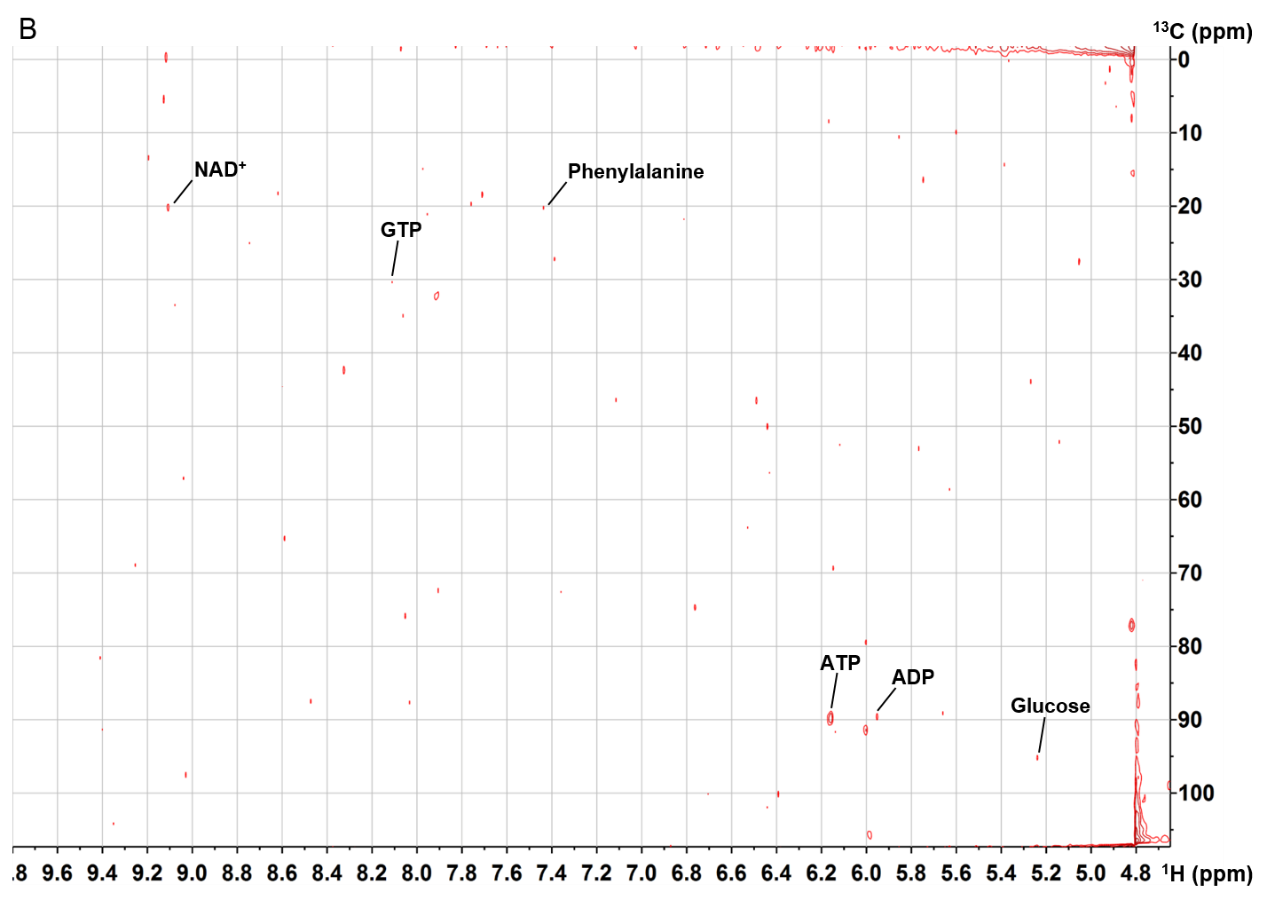


**Supplementary Figure 3. Representative 2D ^1^H-^13^C HSQC spectrum of aqueous extracts derived from C2C12 myoblasts recorded on 850 MHz NMR spectrometer.** Selected regions of 0.0-4.8 ppm **(A)**, 4.8-9.6 ppm **(B)**.

**Supplementary Figure 4**


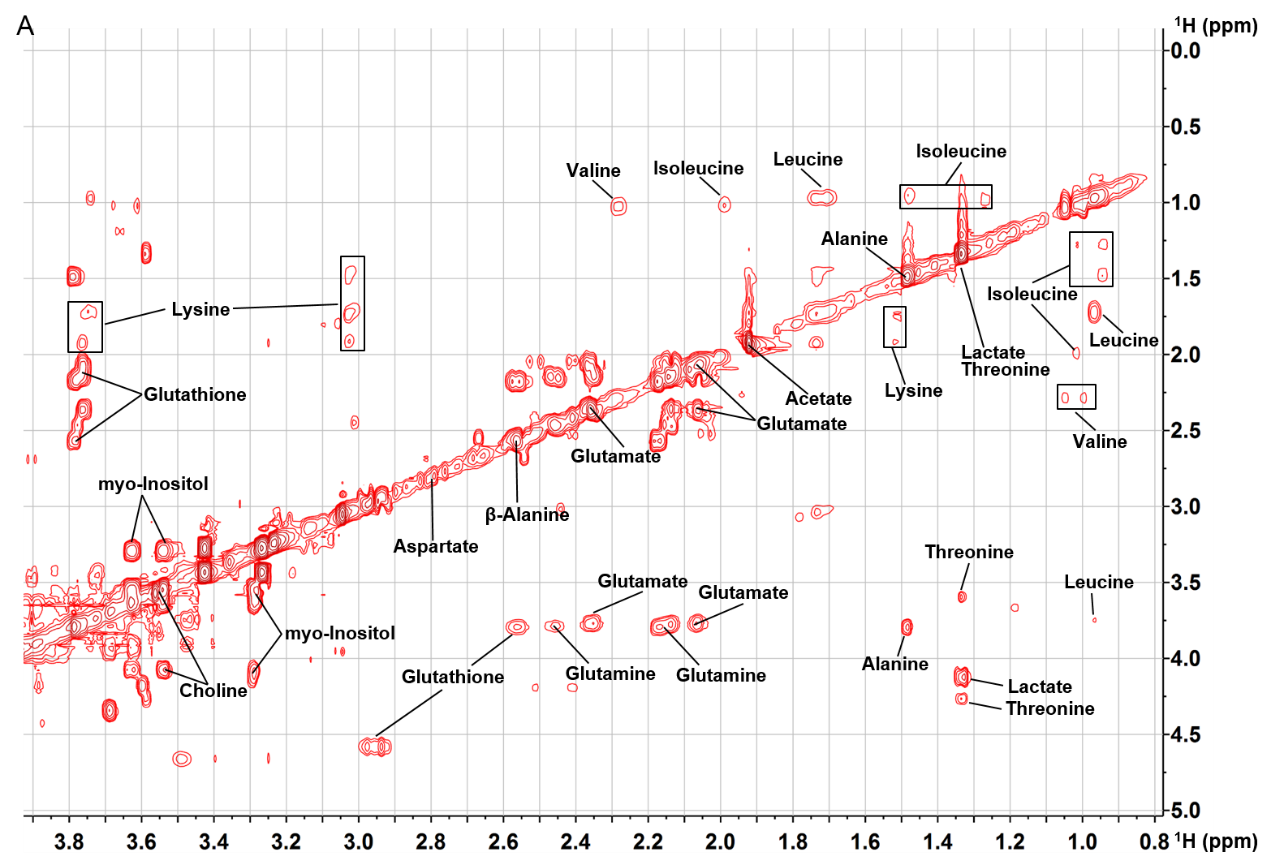


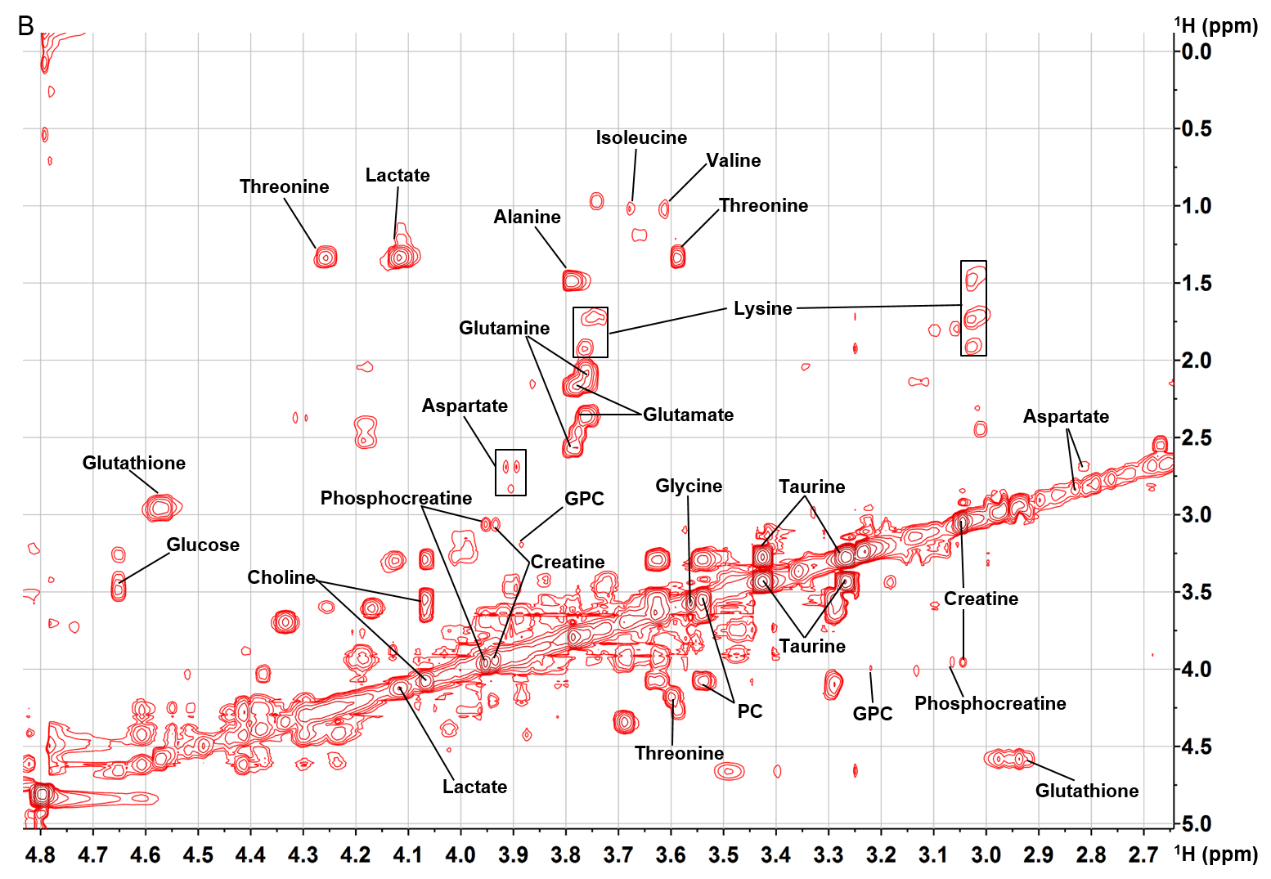


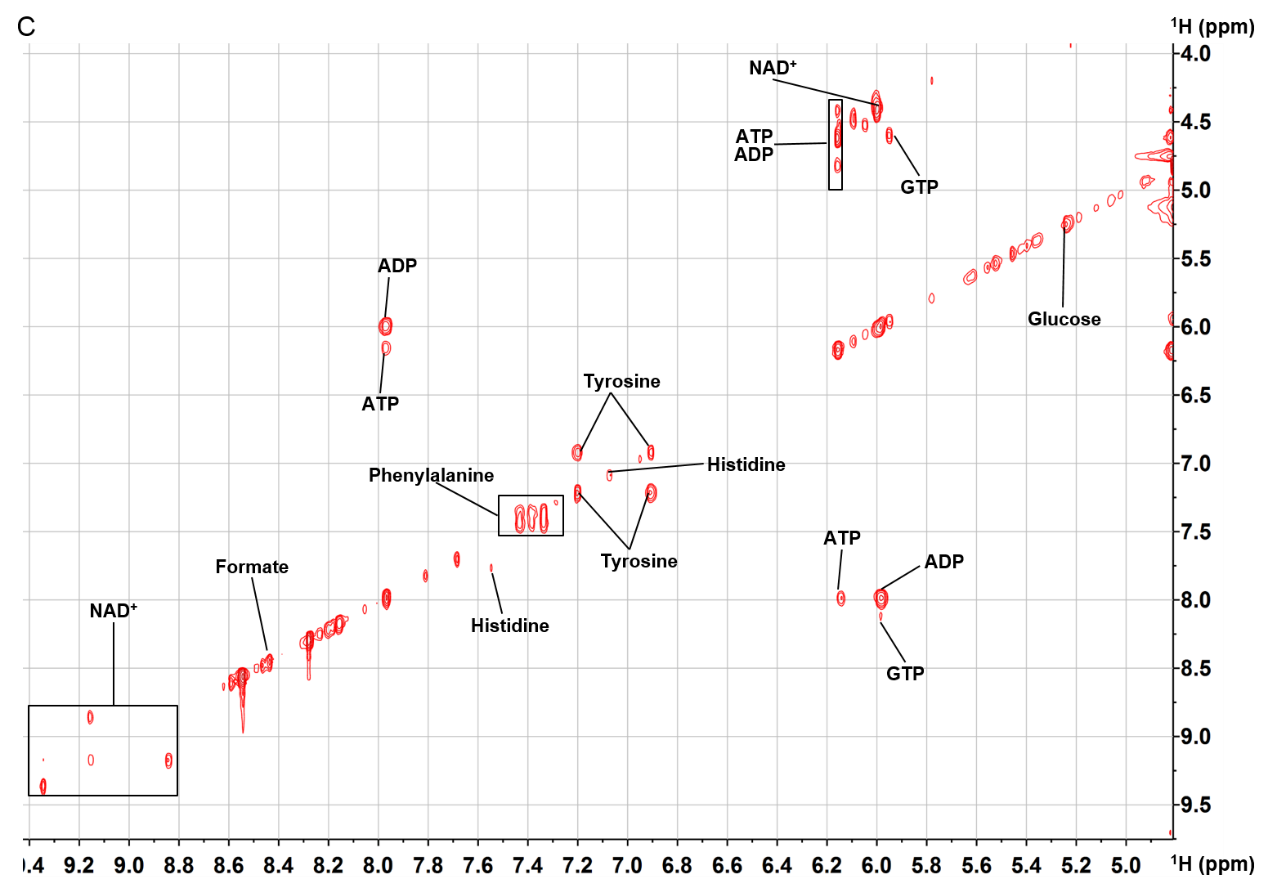


**Supplementary Figure 4.** **Representative 2D ^1^H-^1^H TOCSY spectrum of aqueous extracts derived from C2C12 myoblasts recorded on 850 MHz NMR spectrometer.** Selected regions of 0.8-3.6 ppm **(A)**, 3.6-4.8 ppm **(B)**, 4.8-9.5 ppm **(C)**.

**Supplementary Figure 5**


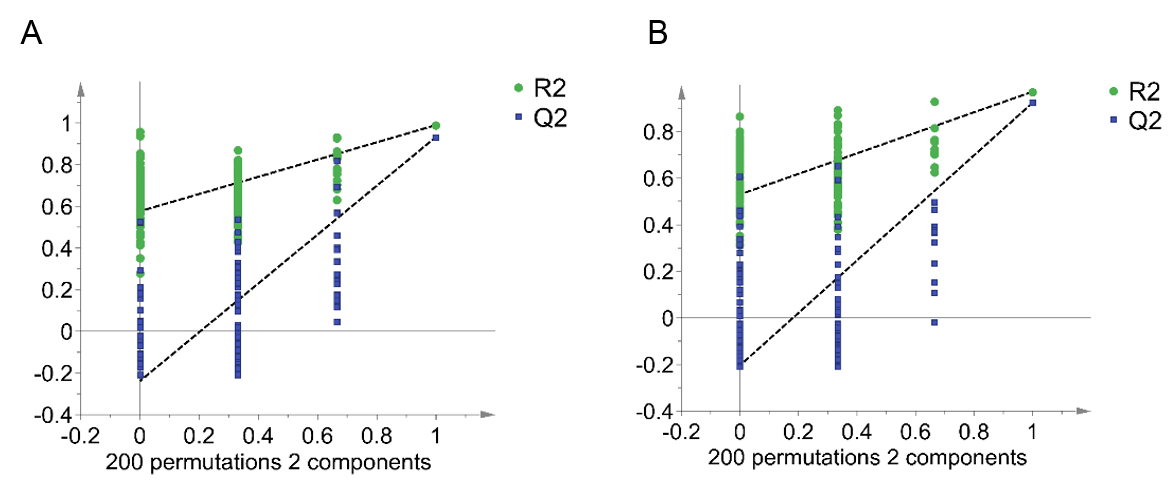


**Supplementary Figure 5. Cross-validation plots of the PLS-DA models for the 1D ^1^H-NMR spectra recorded on aqueous extracts derived from the three groups of C2C12 myoblasts. (A)** CIS *vs.* NOR, **(B)** TAU *vs.* CIS. The cross-validation plots were obtained by response permutation tests using the first two components (n=200).

**Supplementary Figure 6**


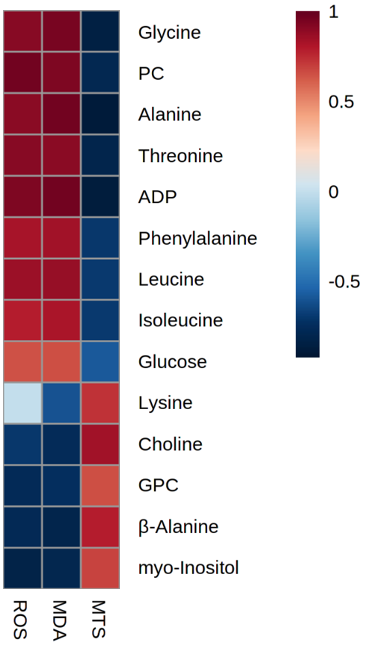


**Supplementary Figure 6. Heat-map exhibits the correlations of intracellular ROS and MDA levels and cell viabilities in CIS myoblasts with intracellular levels of the characteristic metabolite identified from the pairwise comparison of CIS *vs.* NOR.** Red/blue colors denote positive/negative correlations.

**Supplementary Figure 7**


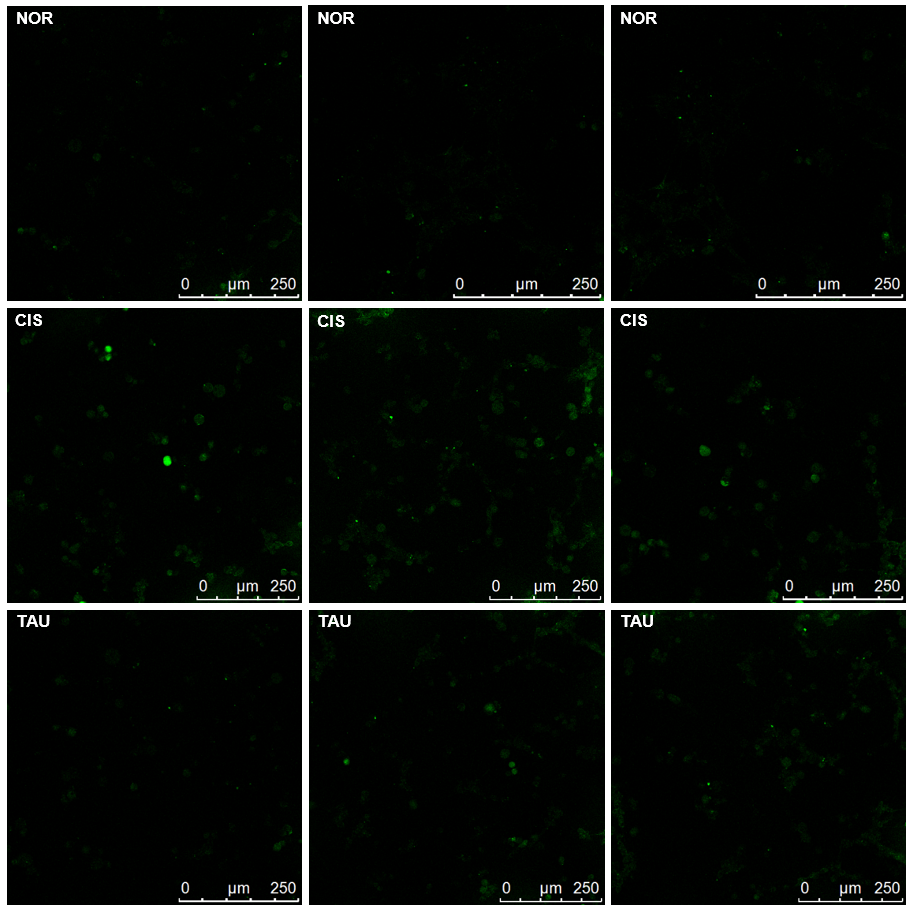


**Supplementary Figure 7. Additional confocal microscopy images of H_2_DCFDA-stained normal control (NOR) myoblasts, cisplatin-treated (CIS) myoblasts, and taurine-supplemented (TAU) myoblasts.** Scale bar, 50 µm.

**Supplementary Figure 8**


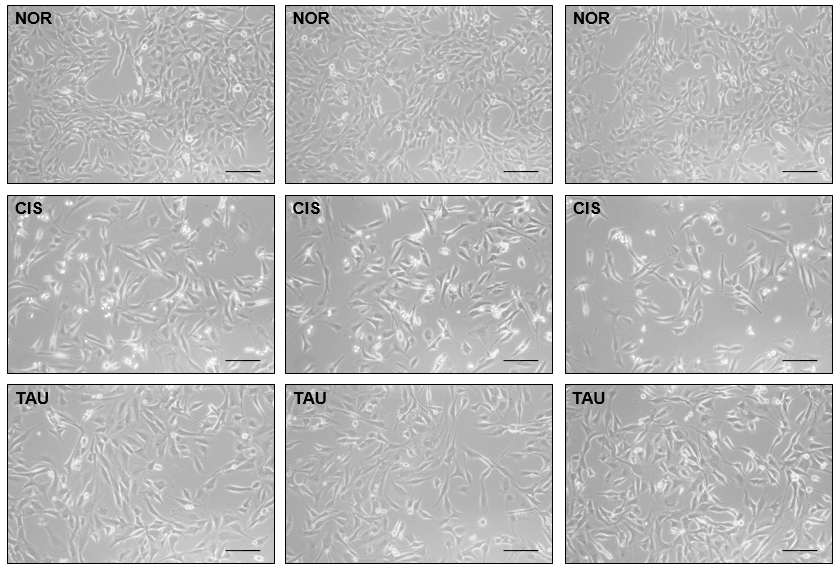


**Supplementary Figure 8. Additional morphological images of the three groups of C2C12 myoblasts (NOR, CIS, and TAU).** Scale bar, 100 μm.

**Supplementary Figure 9**


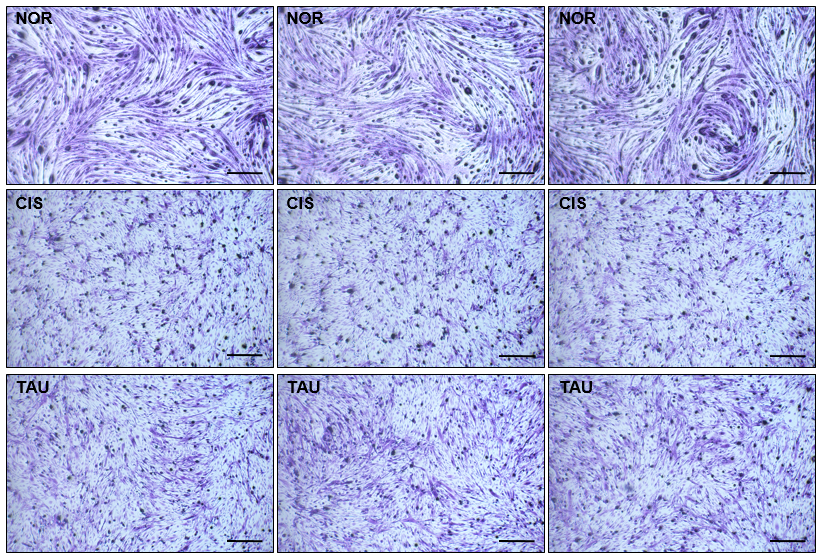


**Supplementary Figure 9. Additional morphological images of normal control (NOR) myotubes, cisplatin-treated (CIS) myotubes and taurine-supplemented (TAU) myotubes (4 ×).** Scale bar, 500 µm.
